# Chemoproteomics unveils Sofalcone targeting ribosomal proteins to inhibit protein synthesis in *Staphylococcus aureus*

**DOI:** 10.1186/s43556-025-00269-4

**Published:** 2025-05-23

**Authors:** Lirun Zhou, Ying Zhang, Ruishen Zhuge, Liqiong Wu, Zheng Chu, Ang Ma, Peng Gao, Yin Kwan Wong, Junzhe Zhang, Xin Peng, Peili Wang, Jigang Wang, Huan Tang

**Affiliations:** 1 State Key Laboratory for Quality Ensurance and Sustainable Use of Dao-Di Herbs, Artemisinin Research Center, and Institute of Chinese Materia Medica, China Academy of Chinese Medical Sciences, Beijing, 100700 China; 2 Department of Pulmonary and Critical Care Medicine, Shenzhen Institute of Respiratory Diseases, Guangdong Provincial Clinical Research Center for Geriatrics, Shenzhen Clinical Research Center for Geriatrics, Shenzhen People’s Hospital, The First Affiliated Hospital, Southern University of Science and Technology, Shenzhen, 518020 Guangdong China; 3 State Key Laboratory of Antiviral Drugs, School of Pharmacy, Henan University, Kaifeng, 475004 China; 4 Peking University School and Hospital of Stomatology, National Center for Stomatology, National Clinical Research Center for Oral Diseases, National Engineering Research Center of Oral Biomaterials and Digital Medical Devices, Beijing, 100081 China; 5 Department of Pathology, Guangzhou First People’s Hospital, Guangzhou, 510180 China; 6 Department of Physiology, National University of Singapore, Singapore, 117543 Singapore; 7 Ningbo Municipal Hospital of TCM Affiliated Hospital of Zhejiang Chinese Medical University, Ningbo, 315010 China; 8 National Clinical Research Center for Chinese Medicine Cardiology, Xiyuan Hospital, China Academy of Chinese Medical Sciences, Beijing, 100091 China

**Keywords:** Sofalcone, *Staphylococcus aureus*, Chemoproteomics, Ribosomal proteins, Antibiotics, Drug-resistance

## Abstract

The escalating threat of antibiotic resistance, particularly in *Staphylococcus aureus* (including methicillin-resistant strains, MRSA), underscores the urgent need for novel therapeutics. Sofalcone (Sof), a chalcone derivative from *Sophora subprostrata* with established anti-inflammatory and anti-ulcer properties, exhibits promising yet underexplored antibacterial activity. Here, we demonstrate that Sof potently inhibits *S. aureus* and MRSA while showing minimal cytotoxicity in human cells. Notably, Sof synergized with amoxicillin, and significantly reduced the pathogenicity of *S. aureus* through inhibiting biofilm formation addressing key virulence factors. Through chemoproteomic profiling using a clickable Sof-derived probe, ribosomal proteins, specifically the 50S subunit protein rplB, were identified as primary targets. Sof covalently binds to rplB via cysteine residues, as validated by cellular thermal shift assays, microscale thermophoresis, and competition assays. Bio-orthogonal noncanonical amino acid tagging revealed that Sof disrupts bacterial protein synthesis by impairing ribosomal function, a mechanism distinct from conventional antibiotics. In a murine model of *S. aureus*-induced acute lung injury, Sof greatly reduced bacterial load in lungs, attenuated systemic inflammation, and mitigated histopathological damage. Its dual antibacterial and anti-inflammatory efficacy, coupled with activity against Gram-negative *Escherichia coli*, highlights broad-spectrum potential. This study unveils a covalent ribosomal-targeting strategy, positioning Sof as a multifaceted candidate against multidrug-resistant infections. Our findings bridge natural product pharmacology and mechanistic antimicrobial discovery, offering a template for combating the global antibiotic resistance crisis.

**Supplementary Information:**

The online version contains supplementary material available at 10.1186/s43556-025-00269-4.

## Introduction

As one of the most notorious and widespread gram-positive bacteria in human and animal populations, *Staphylococcus aureus (S. aureus)* is responsible for numerous infectious diseases, including endocarditis, pneumonia, toxic shock syndrome, sepsis, and biofilm infections, contributing to an increasing annual mortality rate [1]. The stagnation in antibiotic discovery, combined with the rapid proliferation of antibiotic resistance, has made the treatment of these infections increasingly difficult. Methicillin-resistant *S. aureus* (MRSA) has emerged as a particularly concerning variant, causing more deaths annually in the United States than acquired immune deficiency syndrome, with bloodstream infections alone accounting for approximately 20,000 fatalities. The Centers for Disease Control and Prevention (CDC) has classified MRSA as a serious public health threat, underscoring the urgent need for effective therapeutic interventions [2]. While antibiotics such as daptomycin and vancomycin are frequently used to treat MRSA infections, their efficacy in addressing pneumonia is limited due to the risk of fostering additional antibiotic resistance and potential side effects [3, 4]. Furthermore, traditional antibiotic therapies often lead to the selection of resistant bacterial strains, exacerbating the global issue of multidrug resistance. This escalating crisis threatens public health and imposes severe economic burdens [5, 6]. Consequently, the discovery and development of newly antibacterial drugs have become imperative.

Natural products derived from diverse biological sources present a promising alternative to synthetic compounds due to their structural diversity, broad spectrum of targets, and reduced adverse effects [7]. Exploring novel antibacterial drugs from natural sources offers a viable strategy for addressing the growing challenge of bacterial resistance. Natural antimicrobial agents are distinguished by their accessibility, potent bioactivity, and diverse mechanisms of action [8]. Among these, bioactive molecules sourced from plants have played a pivotal role in advancing drug development. For instance, herbs are a rich source of natural compounds with substantial potential for overcoming the current antibiotic resistance crisis [9]. Flavonoids, which are abundant in many plant species, have demonstrated strong antibacterial properties, underscoring their potential as alternative therapeutic agents [10]. Additionally, plant-derived compounds have been shown to synergize with existing antibiotics, enhancing their effectiveness against resistant bacterial pathogens [11]. However, the clinical application of these medicinal plant compounds requires further investigation through double-blind toxicological, rigorous, well-controlled, and clinical studies to ensure their efficacy and safety [12].

Sof, a 2′-carboxymethoxy-4,4′-bis(3-methyl-2-butenyloxy) chalcone, is one of the major derivatives of sophoradin isolated from the root of traditional medical plant *Sophora subprostrata* [13]. Sof demonstrates multifaceted pharmacological efficacy, encompassing antioxidative, gastroprotective, anti-inflammatory, and antimicrobial properties [14]. It has been employed as an anti-ulcer medication in Japan and South Korea for over three decades, owing to its pleiotropic mechanisms of action [15]. Our prior research demonstrated that Sof effectively blocks the interaction between Toll-like receptor 4 (TLR4) and High Mobility Group Protein B1 (HMGB1), suppressing subsequent nuclear factor kappa-B (NF-κB) activation and reducing HMGB1-related pro-inflammatory responses [16]. Additionally, Sof’s anti-ulcer efficacy has been attributed to its inhibition of 15-hydroxy-prostaglandin (PG)-dehydrogenase and its ability to upregulate heme oxygenase (HO)-1 expression in adipocyte and gastric epithelial cells, which enhances mucosal blood flow [17, 18]. Beyond its anti-ulcer properties, Sof has shown potential as an antibacterial agent. Studies have revealed that Sof exerts a direct bactericidal effect against *Helicobacter pylori* (*H. pylori*) by impairing chemotactic motility, adhesion, and vacuolating toxin production [19, 20]. In *H. pylori*-infected mice, Sof significantly ameliorates ulcer formation and reduces bacterial colonization [21]. These findings raise the intriguing question of whether Sof can also combat Gram-positive bacteria, such as *S. aureus*, and the molecular mechanisms underlying its antibacterial activity.

In this study, we performed a systematic evaluation of Sof activity and selectivity against *S. aureus* and drug-resistant strains, as well as its impact on bacterial pathogenicity. To elucidate its mechanism of action, we employed quantitative chemoproteomic profiling to comprehensively identify Sof’s protein targets in *S. aureus* by coupling a chemical probe tagged with a clickable alkyne. Upon confirming ribosomal proteins as the primary targets, we further investigated Sof’s inhibitory effects on protein synthesis in *S. aureus* through bio-orthogonal noncanonical amino acid tagging technique. Finally, we assessed the therapeutic potential of Sof in a mouse model of *S. aureus*-induced acute lung injury (ALI). Our findings indicate that Sof, a natural product derived from medicinal plants, exhibits significant potential as a therapeutic agent for treating *S. aureus* infections and associated diseases. These results provide a foundation for further exploration of Sof as a candidate in the fight against antibiotic-resistant bacterial pathogens.

## Results

### Sofalcone effectively killed bacteria with high cytocompatibility

**Fig. 1:**
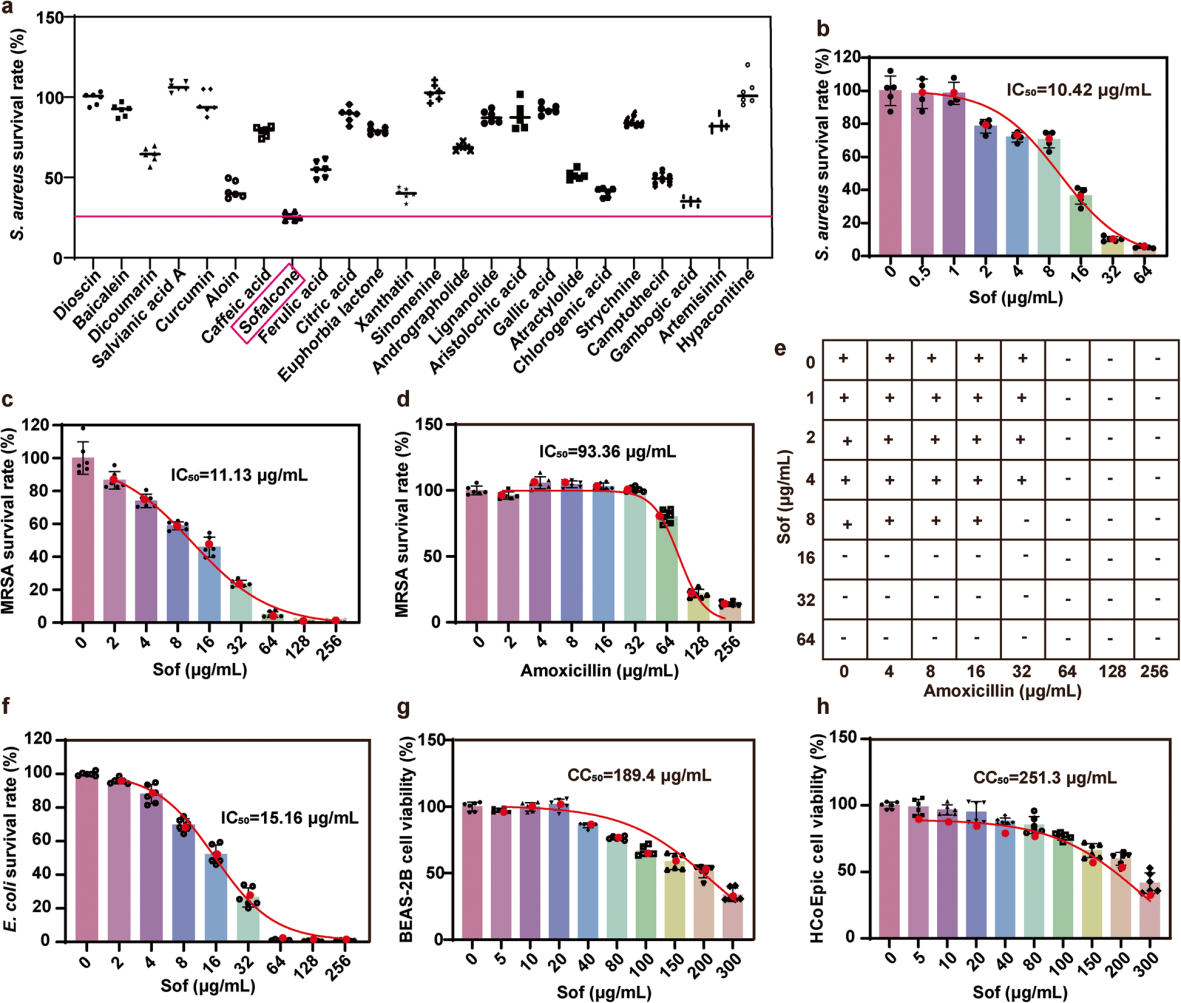
Sof selectively killed bacteria with limited cytotoxicity on human cells*.* **a** Bacterial survival rate of *S. aureus* following 12 h of treatment with natural products at 20 μg/mL (*n* = 6). **b** Bacterial survival rate of *S. aureus* following 12 h of treatment with Sof at different concentrations (*n* = 6). **c** Bacterial survival rate of Methicillin-resistant *S. aureus* (MRSA) following 12 h of treatment with Sofalcone at different concentrations (*n* = 6). **d** Bacterial survival rate of MRSA following 12 h of treatment with amoxicillin at different concentrations (*n* = 6). **e** The checkerboard method showing the synergy of Sof and amoxicillin combination. “ + ”, none- sterile; “-”, sterile. **f** Bacterial survival rate of *E. coli* following 12 h of treatment with Sofalcone at different concentrations (*n* = 6). **g** Cell viability of BEAS-2B cells following 12 h of treatment with Sofalcone at different concentrations (*n* = 6). **h** Cell viability of HCoEpic cells following 12 h of treatment with Sofalcone at different concentrations (*n* = 6)

In an initial small-scale screening of natural products for antibacterial activity against *S. aureus*, Sof emerged as the most promising candidate (Fig. 1a). Subsequently, the germicidal effect of Sof on *S. aureus* and MRSA was evaluated and compared. As shown in Fig. 1b-c, the half-maximal inhibitory concentration (IC_50_) of Sof against *S. aureus* and MRSA was determined to be 10.42 and 11.13 μg/mL respectively, following 12 h of treatment. In contrast, the IC_50_ of amoxicillin against MRSA under the same conditions was significantly higher at 93.36 μg/mL (Fig. 1d). To further investigate the synergistic effect between Sof and amoxicillin, we conducted a checkerboard assay to evaluate the combined antibacterial effect of Sof and amoxicillin (Fig. 1e). Notably, when Sof and amoxicillin were combined, the calculated fractional inhibitory concentration (FIC) index was 0.25, which is below 0.5, indicating a synergistic effect between the two. Given that *S. aureus* is a Gram-positive bacterium, we further evaluated Sof’s antibacterial activity against the Gram-negative bacterium *Escherichia coli* (*E. coli*), with an IC_50_ of 15.16 µg/mL (Fig. 1f). These findings highlight Sof’s potential as a broad-spectrum antibacterial agent. To assess its selectivity, the cytotoxicity of Sof was tested in human lung epithelial cells (BEAS-2B) and human colonic epithelial cells (HcoEpic). Cell viability assays revealed 50% cytotoxic concentrations (CC_50_) of 189.4 µg/mL and 251.3 µg/mL for BEAS-2B and HcoEpic cells, respectively, both of which are significantly higher than the minimum inhibitory concentration (MIC) of 64 µg/mL for Sof against *S. aureus* and MRSA (Figs. 1g-h). Collectively, these results confirm that Sof selectively and effectively kills bacteria while exhibiting minimal cytotoxicity to human cells.

**Fig. 2:**
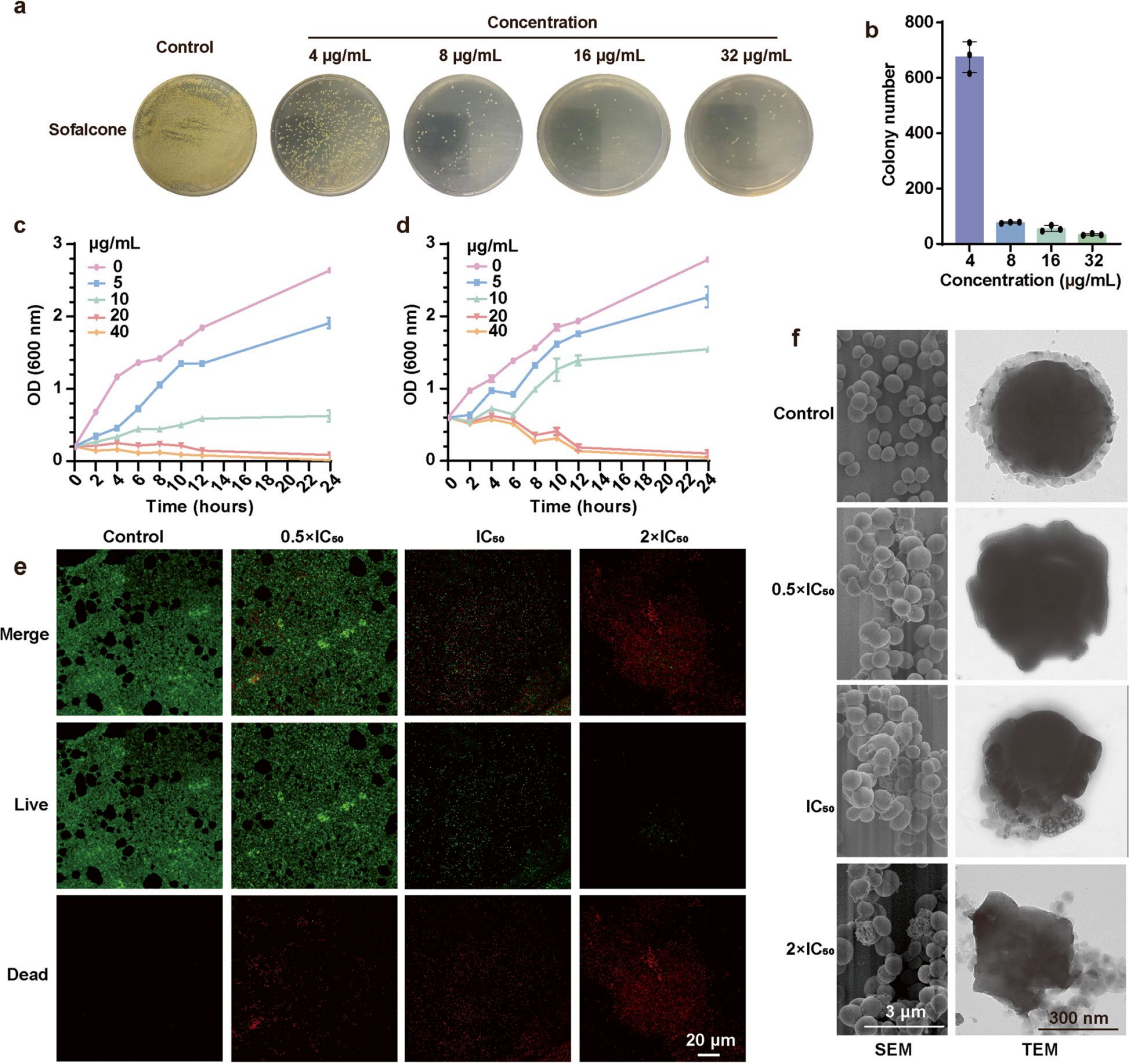
Sof effectively inhibited the growth of *S. aureus*. **a** Optical images of *S. aureus* colony following 6 h of treatment with Sofalcone at different concentrations. **b** Colony counting number of *S. aureus* following 6 h of treatment with Sofalcone at different concentrations (*n* = 3). **c** Growth curve of *S. aureus* within 24 h of treatment with Sof at different concentrations (*n* = 3). **d** Killing curve of *S. aureus* within 24 h of treatment with Sofalcone at different concentrations (*n* = 3). **e** Fluorescence imaging showing the live and dead status of *S. aureus* following 6 h of treatment with Sof at different concentrations. Live and dead cells are indicated by green and red fluorescence, respectively. **f** Morphological characterization of *S. aureus* following 6 h of treatment with Sof at different concentrations by scanning electron microscope (SEM) and transmission electron microscope (TEM)

To further investigate the antibacterial effects of Sof against *S. aureus*, a plate colony count assay was conducted (Fig. S1) [22]. The results demonstrated a concentration-dependent reduction in bacterial colony numbers (Fig. 2a-b), consistent with the MIC data. Additionally, growth curve analysis revealed that Sof inhibited bacterial growth in a dose-dependent manner, with *S. aureus* growth nearly abolished at a concentration of 20 µg/mL (Fig. 2c). The bacterial killing curve further indicated that Sof at concentrations of 20 µg/mL and 40 µg/mL eliminated most bacteria within 12 h (Fig. 2d). These findings, supported by MIC and minimum bactericidal concentration (MBC) data, underscore Sof’s effectiveness as a bactericidal agent. Live and dead fluorescence staining assays corroborated the potent lethality of Sof against *S. aureus* (Fig. 2e). Morphological changes in *S. aureus* following Sof treatment were investigated using transmission electron microscopy (TEM) and scanning electron microscopy (SEM). Untreated bacteria exhibited smooth and intact surfaces, whereas Sof-treated cells displayed irregular, collapsed structures, indicating severe cellular damage (Fig. 2f). In summary, Sof demonstrates robust antibacterial activity, excellent cytocompatibility, and remarkable effectiveness against *S. aureus* and MRSA, making it a promising candidate for further therapeutic development.

### Sofalcone significantly reduced the pathogenicity of *S. aureus*

**Fig. 3:**
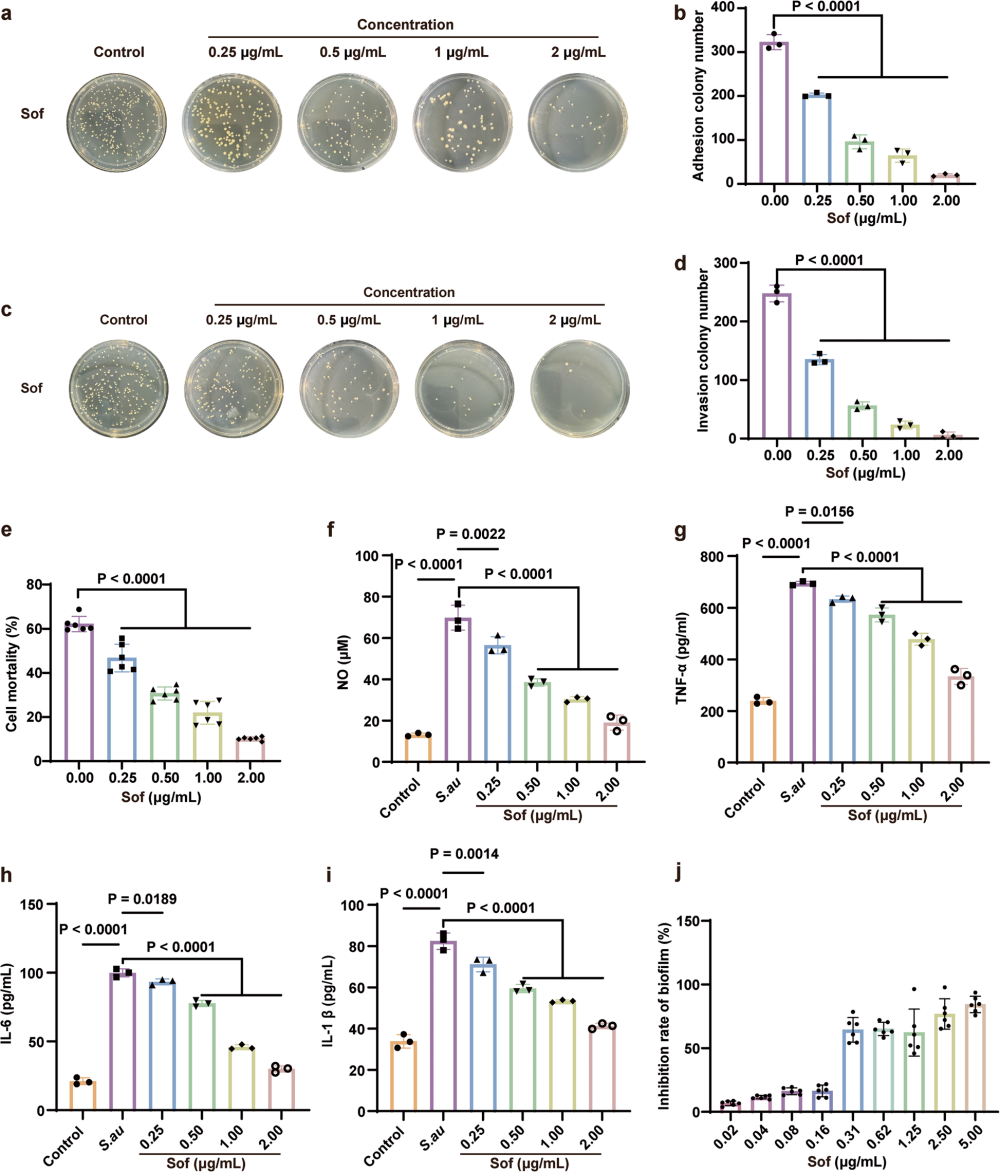
Sof significantly reduced the pathogenicity of *S. aureus.* **a** Optical images of *S. aureus* colonies adhering to BEAS-2B cells following 6 h of treatment with Sofalcone at different concentrations (*n* = 3). **b** Colony count of *S. aureus* adhering to BEAS-2B cells following 6 h of treatment with Sofalcone at different concentrations (*n* = 3). **c** Optical images of *S. aureus* colonies invading BEAS-2B cells following 6 h of treatment with Sofalcone at different concentrations (*n* = 3). **d** Colony count of *S. aureus* invading BEAS-2B cells following 6 h of treatment with Sofalcone at different concentrations (*n* = 3). **e** Cell mortality of BEAS-2B cells after *S. aureus* infection following 6 h of treatment with Sofalcone at different concentrations (n = 3). **f-i** Secretion of NO (**f**), TNF-α (**g**), IL-6 (**h**), and IL-1β (**i**) by BEAS-2B cells after *S. aureus* infection following 6 h of treatment with Sofalcone at different concentrations (*n* = 3). **j** Biofilm formation of *S. aureus* following 6 h of treatment with Sofalcone at different concentrations (*n* = 6). Data are expressed as the mean ± SEM

The pathogenicity of *S. aureus* is central to its ability to cause infectious diseases and is primarily characterized by its capacity to form biofilms, adhere to and invade host cells, and produce toxins and inflammatory factors [23–25]. Biofilm formation is a key driver of persistent infections and antibiotic resistance, while the virulence of *S. aureus* is closely linked to its adhesion and invasion capabilities [26, 27]. To evaluate the impact of Sof on *S. aureus* pathogenicity, invasion and adhesion assays were performed. As expected, Sof significantly reduced the adhesion (Figs. 3a-b) and invasion (Figs. 3c-d) of *S. aureus* to BEAS-2B cells in a dose-dependent manner, as evidenced by a marked decrease in the bacterial colony counts after adhesion and invasion. Furthermore, cell mortality induced by *S. aureus* was substantially alleviated in the presence of Sof in a concentration-dependent manner (Fig. 3e). We further assessed the impact of Sof on inflammatory responses triggered by *S. aureus* in BEAS-2B cells. The secretion of inflammatory cytokines, including tumor necrosis factor-alpha (TNF-α), nitric oxide (NO), interleukin-1 beta (IL-1β), and interleukin-6 (IL-6), was significantly elevated following *S. aureus* exposure. However, Sof markedly mitigated these inflammatory responses in a dose-dependent manner (Figs. 3f-i). Additionally, crystal violet staining revealed that Sof effectively inhibited biofilm production by *S. aureus*. The biofilm inhibition rate reached 84% at a Sof concentration of 5 µg/mL (Fig. 3j). Collectively, these findings indicate that Sof significantly reduced the pathogenicity of *S. aureus* by suppressing invasion, adhesion, and biofilm formation capabilities, while also ameliorating inflammation and cellular toxicity induced by *S. aureus*.

### Chemoproteomic revealed protein targets of Sofalcone in *S. aureus*

**Fig. 4:**
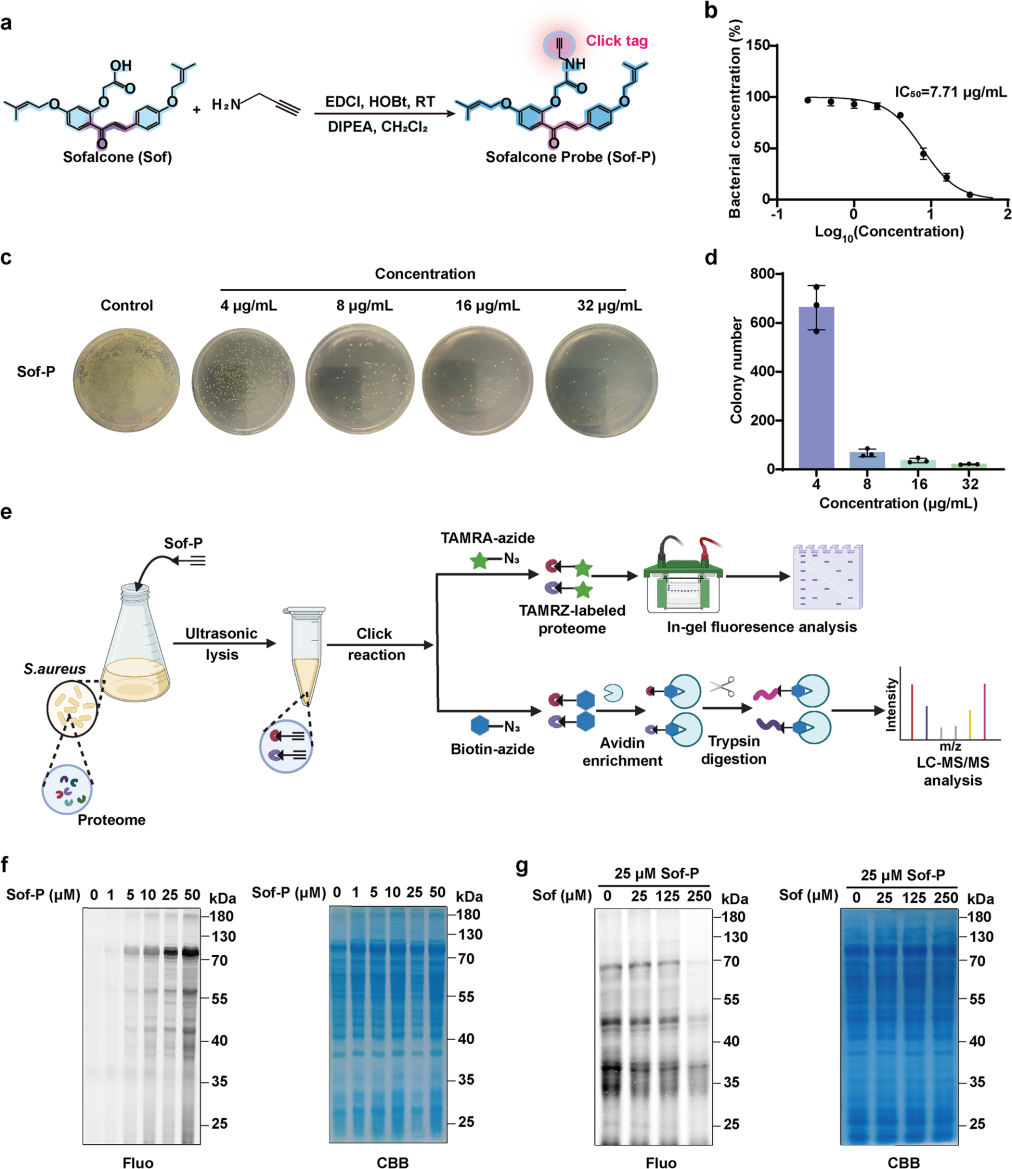
Antibacterial effect of Sof-P against *S. aureus* and the proteome labeling efficiency. **a** Synthesis scheme of sofalcone probe bearing alkynyl (Sof-P). **b** The half inhibitory concentrations (IC_50_) of Sof-P against *S. aureus* after 12 h of treatment. **c** Optical images of *S. aureus* colony following 6 h of treatment with Sof-P at different concentrations. **d** Colony counting number of *S. aureus* following 6 h of treatment with Sof-P at different concentrations (*n* = 3). **e** Overall workflow for profiling protein targets of Sof in *S. aureus*. **f** Fluorescent labelling of proteins in *S. aureus* by Sof-P after in situ treatment. Coomassie brilliant blue (CBB) staining was used to normalize the amount of whole protein. **g** Competitive fluorescent labelling of proteins in *S. aureus* by Sof-P after in situ treatment in the presence of excess Sof. Coomassie brilliant blue (CBB) staining was used to normalize the amount of whole protein

Next, we sought to elucidate the detailed molecular mechanisms underlying the antibacterial activity of Sof. Given that Sof contains a 1,3-diaryl-2-propen-1-one (chalcone) structure, which includes an electrophilic functional group acting as a Michael reaction acceptor (α, β-unsaturated carbonyl system), we planned to employ an activity-based protein profiling (ABPP) assay. This approach aimed to identify covalent protein targets through potential in situ bioactivation or direct covalent reaction [28, 29]. To this aim, we designed and successfully synthesized a chemical probe of Sof (Sof-P), which bears a clickable alkyne reactive handle, using a one-step synthetic route (Fig. 4a and Fig. S2-S3. The IC_50_ of Sof-P was measured at 7.71 μg/mL, indicating that the chemical probe exhibits similar or even better antibacterial effects compared to the parent compound (Fig. 4b and Fig. S4). Meanwhile, Sof-P also showed a dose-dependent effect in reducing colony counting number of *S. aureus* (Fig. 4c-d).

Subsequently, we employed a chemical proteomics strategy to globally profile the protein targets of Sof in *S. aureus*, following the workflow outlined in Fig. 4e. To make sure the protein amount was consistent across the groups before fluorescent labeling and proteomics identification, the proteins exacted from *S. aureus* in different groups were quantified through bicinchoninic acid (BCA) assay, followed the normalization to the same concentration. The proteins captured by Sof-P were directly visualized after conjugation with a fluorescent dye through copper(I)-catalyzed azide-alkyne cycloaddition (CuAAC) click chemistry. As anticipated, the fluorescence intensity of proteins labeled by Sof-P increased in a dose-dependent manner, suggesting that Sof-P could covalently engage with proteins (Fig. 4f). Additionally, in situ pre-incubation with Sof significantly competed away the labeling of Sof-P in *S. aureus*, indicating that Sof-P and Sof shared the same protein targets (Fig. 4g).

**Fig. 5:**
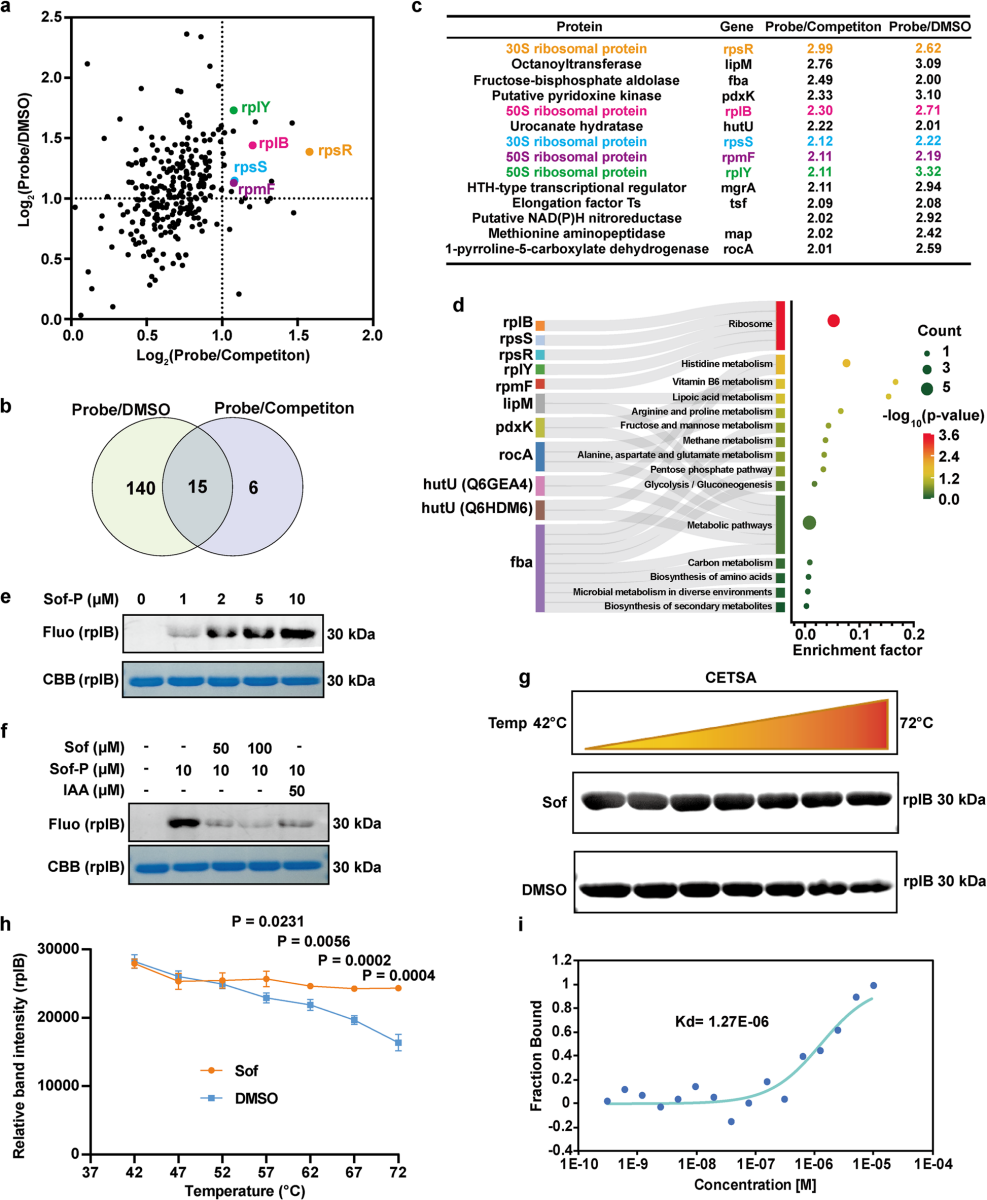
Protein target identification of Sof in *S. aureus* via quantitative chemoproteomic profiling. **a** Volcano plot depicting differential protein profiles captured in the Sof-P group versus the DMSO and competition groups. **b** Venn diagram displaying the number and overlap of protein targets in the Sof-P group versus the DMSO and competition groups **c** The table list including the protein name and the quantitative ratios of the 15 overlapped proteins. **d** KEGG pathway enrichment analysis of Sof-targeted proteins. **e** Fluorescence labeling of the Sof probe on purified recombinant rplB protein (Sof 1 μM = 0.45 μg/mL). **f** Competition assay of the Sof probe on purified recombinant rplB protein in the presence of Sof and IAA. **g**-**h** Cellular thermal shift assays showing the thermal ability of rplB in the absence and presence of Sof (13 μM). **i** Binding affinity of rplB for Sof, measured using microscale thermophoresis (MST)

To further identify these protein targets, the probe-labeled proteins were conjugated with biotin through click chemistry and enriched using streptavidin affinity purification, followed by trypsin digestion. Tandem mass tag (TMT)-based quantitative proteomic analysis was then employed to quantify the protein ratios [30]. The relative aboundance ratios of proteins enriched by Sof-P in the control group (Sof-P vs DMSO) and the competition group (Sof-P vs Sof-P + Sof) were calculated to screen out the hit proteins (Table S1). By applying a cut-off of 2.0 for the ratios and 0.05 for the p-values, volcano plots revealed 21 targets in the competition group (Fig. S5a) and 155 targets in the control group (Fig. S5b), respectively. To eliminate false positives from nonspecific binding, we analyzed the overlap between the two groups and identified 15 qualified protein targets that covalently bound to Sof (Fig. 5a-b). Interestingly, five of these 15 protein targets were ribosomal proteins, ranking within the top 10 ratios in the competition group (Fig. 5c). These 15 potential targets were further subjected to bioinformatics analysis to identify the affected molecular pathways. Gene ontology (GO) pathway analysis indicated that these targets were primarily enriched in the ribosomal subunit and ribonucleoprotein complex (Fig. S6). Additionally, Kyoto Encyclopedia of Genes and Genomes (KEGG) analysis suggested that these targets of Sof were involved in multiple biological processes, including ribosome function, amino acid metabolism, and glycolysis (Fig. 5d). Collectively, chemical proteomics revealed that ribosomal proteins were the high-priority and confident targets of Sof in *S. aureus*.

To validate Sof's interaction with ribosomal proteins, the 50S ribosomal protein (rplB) was overexpressed and purified for further analysis. Sof-P labeling of recombinant rplB was concentration-dependent and was effectively competed by excess Sof or iodoacetamide (IAA), a cysteine-blocking agent, indicating covalent binding to rplB via cysteine conjugation (Fig. 5e-f). Cellular thermal shift assay (CETSA) demonstrated enhanced thermal stability of rplB in the presence of Sof, supporting direct interaction (Fig. 5g-h). Microscale thermophoresis (MST) analysis further quantified the binding affinity, revealing a dissociation constant (Kd) of 1.27 µM (Fig. 5i). These results collectively demonstrate that Sof targets ribosomal proteins such as rplB through cysteine-dependent covalent conjugation, with strong binding affinity, thereby elucidating the molecular basis of its antibacterial activity.

### Sofalcone suppressed protein synthesis by targeting ribosomal proteins

**Fig. 6:**
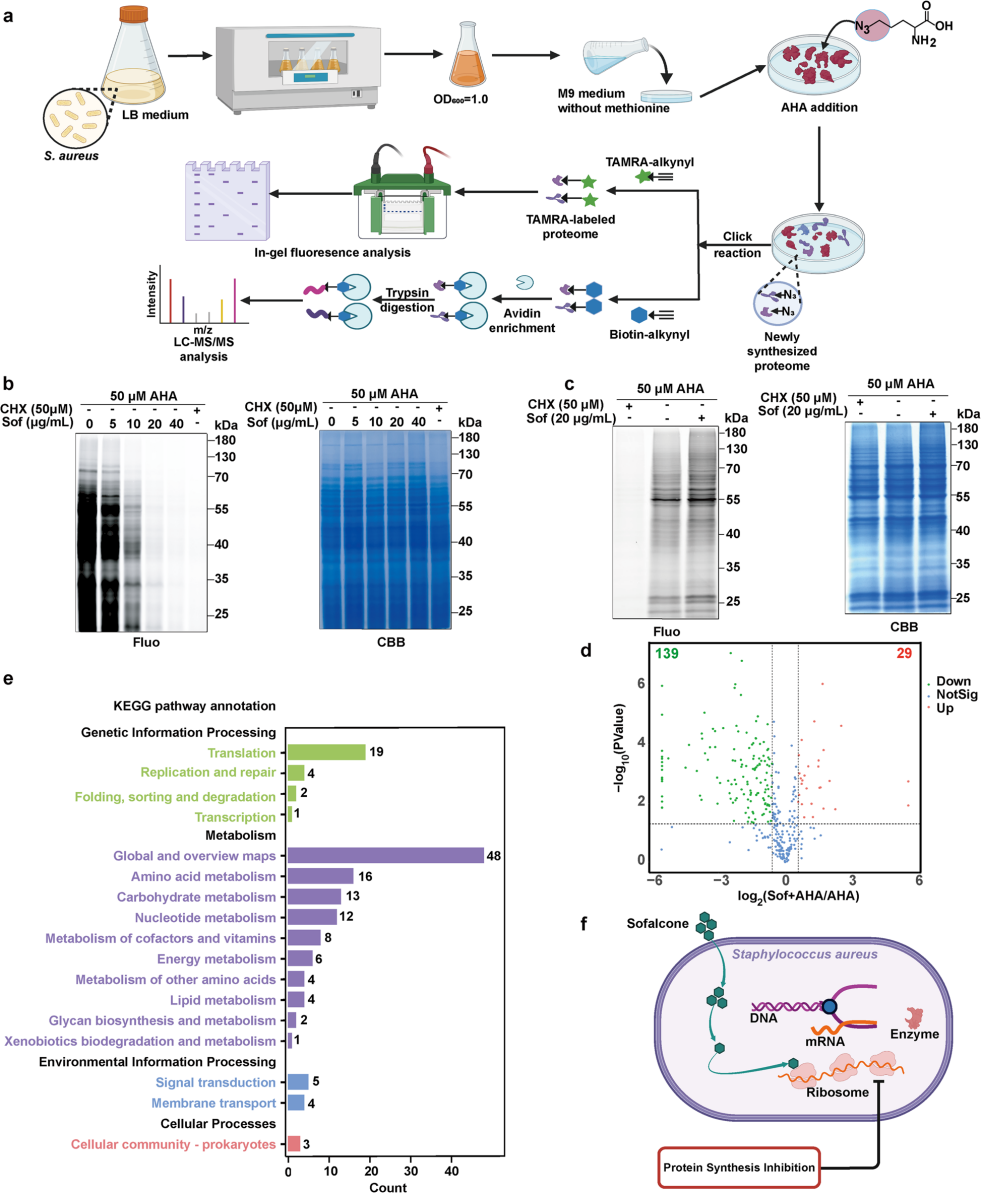
Sof suppressed protein synthesis in *S. aureus* by targeting ribosome proteins. **a** Schematic workflow for detecting newly synthesized proteins in *S. aureus* through AHA metabolic labelling. **b** Fluorescence labeling of proteins bearing AHA incorporation in *S. aureus* in the presence of Sof at different concentrations. **c** Fluorescence labeling of proteins bearing AHA incorporation in BEAS-2B cells in the absence and presence of Sof. **d** Volcano plot for differential protein profiles in the groups of AHA + Sof versus AHA. **e** KEGG pathway enrichment analysis of proteins whose synthesis inhibited by Sof. **f** Illustration of mechanism of actions of Sof against *S. aureus* by hampering protein synthesis

The ribosome, an evolutionarily conserved universal machinery, orchestrates protein synthesis through translation, a central biological process indispensable for cellular function across all life forms. [31, 32]. The synthesis of new proteins is essential for maintaining the life activities of an organism [33]. In prokaryotes, each protein, regardless of its function or origin, is synthesized through mRNA-templated translation on ribosomes. Once synthesized, emerging polypeptide chains immediately begin folding into their unique three-dimensional (3D) structures to become biologically active [34]. Given that ribosomal proteins have been identified as direct targets of Sof in *S. aureus*, we next investigated whether Sof could suppress the process of protein synthesis, thereby killing the bacterium. To test this hypothesis, we employed bio-orthogonal noncanonical amino acid tagging (BONCAT) technology to label and detect newly synthesized proteins. This method involves incorporating a methionine analogue, L-azidohomoalanine (AHA), into proteins through normal protein translation mechanisms in living organisms, without causing measurable adverse effects on their functions [35]. To ensure accurate AHA incorporation, methionine was removed from the culture medium for *S. aureus* in advance. Newly synthesized AHA-tagged proteins were then selectively visualized by in-gel fluorescence imaging or identified by LC–MS/MS after being conjugated to a fluorophore or biotinylated tag via CuAAC click chemistry (Fig. 6a) [36, 37]. This strategy has been successfully used to monitor dynamic changes in the amount and content of newly synthesized proteins in prokaryotes such as *E. coli* [38].

After selecting a culture concentration of AHA at 50 µM based on the literature [39], we investigated the inhibitory effects of Sof on protein synthesis using BONCAT technology. In the absence of Sof, the fluorescence intensity of proteins increased with incubation time, reaching a plateau at 60 min. However, in the presence of Sof, the labeling intensity was significantly reduced, indicating that Sof impeded AHA incorporation into proteins by inhibiting the protein synthesis process (Fig. S7). Furthermore, higher concentrations of Sof led to a greater decrease in the intensity of labeled proteins, with the inhibitory effects reaching a plateau at 20 µg/mL (Fig. 6b). The addition of cycloheximide (CHX), a known protein synthesis inhibitor, produced suppression effects similarly to those of Sof. These findings collectively indicate that Sof inhibits protein synthesis in a concentration-dependent manner by targeting ribosomal proteins in *S. aureus*. To further assess whether Sof affects protein synthesis in mammalian cells at this concentration, we conducted the BONCAT experiment using BEAS-2B cells (Fig. 6c). The results showed that Sof selectively inhibited protein synthesis in *S. aureus* without affecting mammalian cells. These results confirm that Sof does not impact protein synthesis in normal human cells at concentrations effective against bacteria.

To further investigate which specific protein synthesis processes were inhibited by Sof treatment, we enriched the proteins in the AHA and Sof + AHA groups using the biotin-avidin system, followed by analysis and quantification via LC–MS/MS. In total, 354 newly synthesized proteins were identified. A volcano plot revealed that 139 proteins were significantly downregulated in the Sof + AHA group compared to the AHA group (Fig. 6d and Table S2). The downregulated proteins were then subjected to bioinformatic analysis. KEGG pathway analysis indicated that these downregulated proteins were enriched in translation processes and amino acid, carbohydrate, and nucleotide metabolism (Fig. 6e). Additionally, GO analysis highlighted significant categories, including organonitrogen compound biosynthetic and metabolic processes, binding, and cytoplasmic components, under the cellular component categories, molecular function and biological process, (Fig. S8). These findings collectively suggest that Sof exerts its bactericidal effect by targeting ribosomal proteins, thereby disrupting protein synthesis (Fig. 6f). This action effectively suppresses vital metabolic pathways and protein translation in *S. aureus* while sparing normal human cells at antibacterial concentrations.

### Sofalcone ameliorated ALI in mice induced by *S. aureus*

**Fig. 7:**
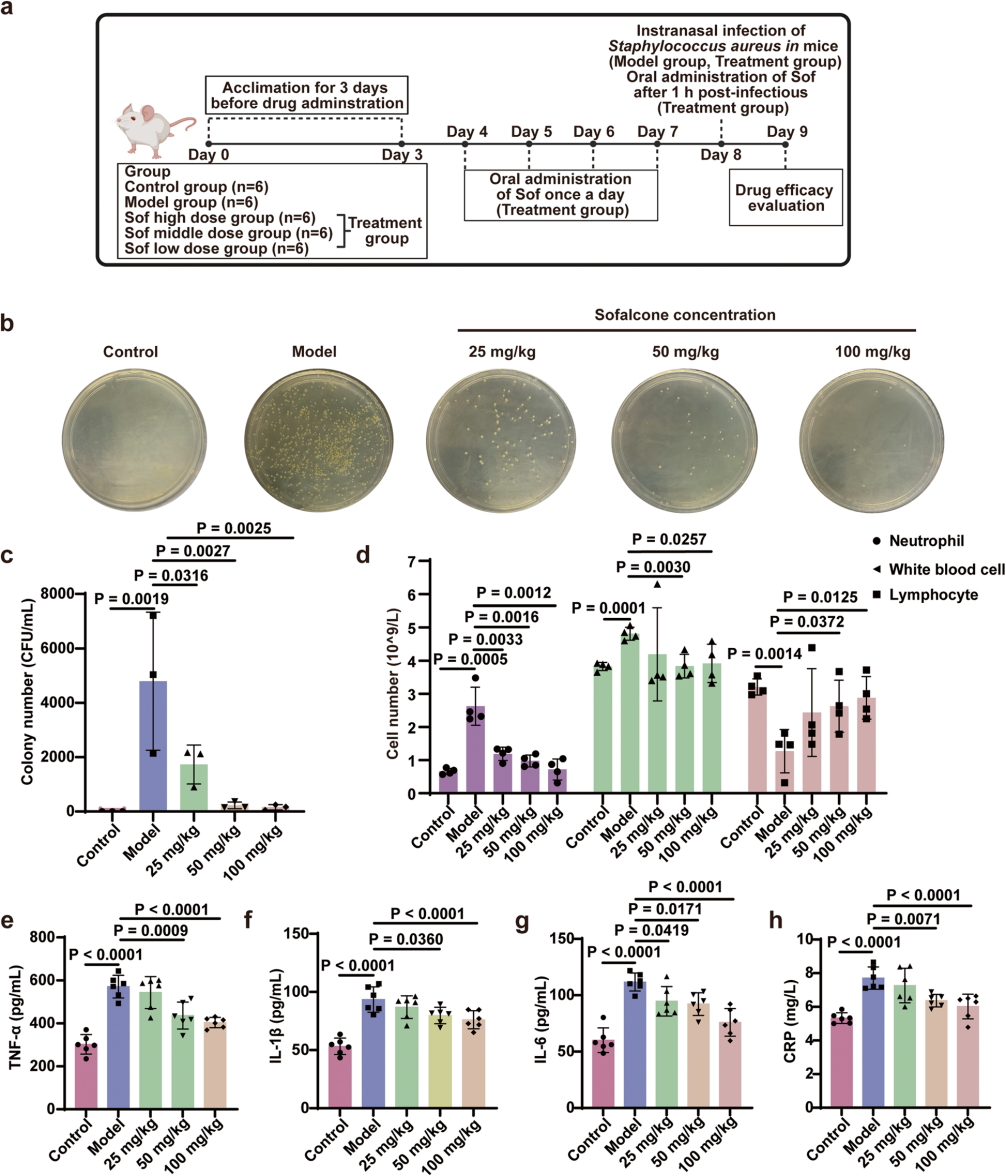
Sof inhibited bacterial reproduction and reduced inflammation level in *S. aureus* infected mice. **a** Schematic illustration of the animal experiment. (Mice were divided into five groups: Control, Model, and Sof treatment group (25, 50, and 100 mg/kg). **b**-**c** In vivo antimicrobial activity assessment of Sof in *S. aureus*-infected mice by plate colony counting method, where the samples were collected from bronchoalveolar lavage fluid (*n* = 3). **d** Inflammatory cells including neutrophil, white blood cell, and lymphocyte counted in peripheral anticoagulation blood (*n* = 4). **e**–**h** Serum level of TNF-α, IL-1β, IL-6, and CRP in mice (*n* = 6). Data are expressed as the mean ± SEM

Bacterial infection is the primary cause of ALI and acute respiratory distress syndrome (ARDS). ALI is characterized by an exaggerated inflammatory response in the lungs, damage to the alveolar capillary barrier, infiltration of neutrophils, lymphocytopenia, and impaired gas exchange due to pulmonary edema [40]. To comprehensively evaluate the pharmacological effects of Sof on *S. aureus*-induced ALI in vivo, we conducted animal experiments according to the schedule outlined in Fig. 7a. To simulate clinical scenarios, Sof was orally administered daily to mice pre-emptively, starting four days before infection. Subsequently, we established an ALI model in BALB/c mice infected with *S. aureus* [41, 42]. Specifically, Sof was administered to mice via gavage one hour after intranasal administration of *S. aureus*. The number of *S. aureus* colonies was subsequently assessed by plating diluted bronchoalveolar lavage fluid (BALF) on medium. As anticipated, Sof reduced *S. aureus* colonies in a dose-dependent manner compared to the model group (Fig. 7b-c). This suggests that Sof could not only kill *S. aureus *in vitro but also greatly inhibit the growth of *S. aureus* in lung tissue in vivo. Moreover, Sof administration dose-dependently reduced the numbers of neutrophils and total white blood cells, as well as the percentage of neutrophils, while concurrently enhancing lymphocyte counts in peripheral blood, implying the improved immune responses by Sof treatment (Fig. 7d and Fig. S9).

**Fig. 8:**
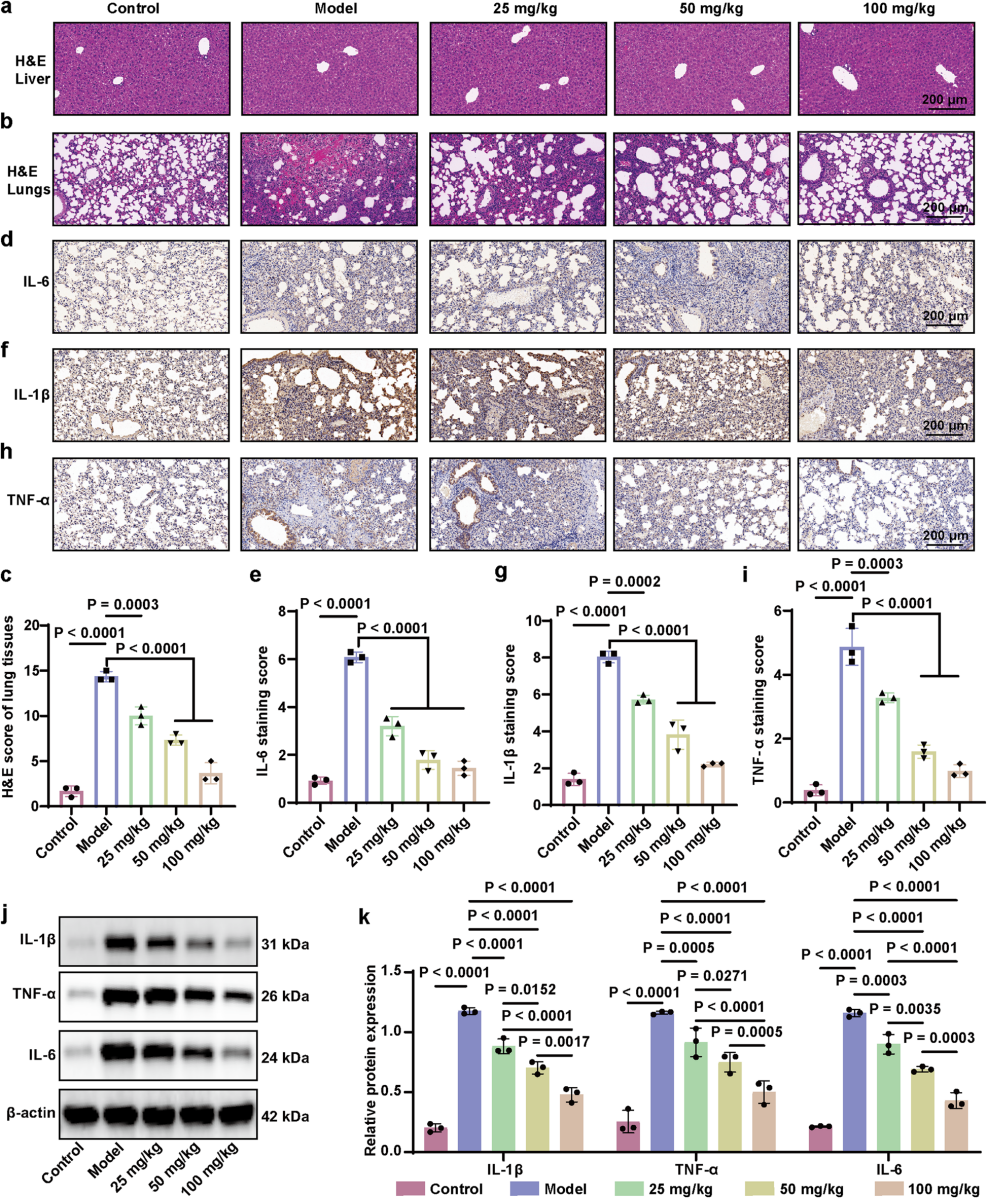
Sof ameliorated acute lung injury in mice induced by *S. aureus*. **a**-**b** Representative hematoxylin and eosin (H&E) staining of liver (**a**) and lung (**b**) tissues. **c** Quantitative scoring of lung histopathological changes (*n* = 3). **d**-**i** Immunohistochemical staining (**d**, **f**, **h**) and staining scores (**e**, **g**, **i**) for IL-6 (**d**-**e**), IL-1β (**f**-**g**), and TNF-α (**h**-**i**) in lung tissues. **j**-**k** Relative expression level of IL-1β, TNF-α, and IL-6 in lung tissues examined by western blot. Data are expressed as the mean ± SEM

*S. aureus* infections are characterized by the excessive production of inflammatory mediators, such as IL-1β, TNF-α, and IL-6, which lead to significant neutrophil infiltration, along with other immune and inflammatory cells [43, 44]. Besides, C-reactive protein (CRP), a highly sensitive inflammatory marker, exhibits elevated concentrations in response to the severity of infection [45]. Therefore, enzyme-linked immunosorbent assay analysis was used to measure the serum levels of these inflammatory markers. As depicted in Fig. 7e-h, Sof dose-dependently decreased the plasm levels of CRP, TNF-α, IL-6, and IL-1β induced by *S. aureus*. Next, we examined the injury and inflammatory responses in lung tissues to further evaluate Sof’s therapeutic efficacy. Hematoxylin and eosin (H&E) staining revealed that Sof treatment markedly alleviated *S. aureus*-induced lung injury, including neutrophil infiltration, thickening of the alveolar walls, and increased lymphocyte infiltration, while showing no observable pathological changes in liver tissue (Fig. 8a-c). Immunohistochemical analysis of lung tissue confirmed that Sof treatment reduced IL-6 (Fig. 8d-e), IL-1β (Fig. 8f-g), and TNF-α (Fig. 8h-i) expression in a dose-dependent manner. These findings were further supported by Western blotting, where Sof treatment effectively suppressed the elevated expression of IL-1β, TNF-α, and IL-6 induced by *S. aureus* infection (Fig. 8j-k). In summary, these experimental findings indicate that Sof effectively ameliorated ALI and the related inflammatory responses caused by *S. aureus*, in a dose-dependent manner.

## Discussion

In recent years, the emergence of antibiotic resistance, particularly against *S. aureus*, has posed a significant challenge to public health [46]. This bacterial pathogen is responsible for a variety of infections, ranging from mild skin conditions to severe systemic diseases such as ALI, sepsis, and pneumonia. The increasing prevalence of MRSA further complicates treatment options, necessitating the development of new therapeutic agents with unique mechanisms of action [47]. Natural products have re-emerged as promising antimicrobial candidates owing to their intrinsic antimicrobial polypharmacology and enhanced biocompatibility compared to synthetic counterparts. This study explores the antibacterial potential of Sof, a natural compound with promising therapeutic properties. The results of our study suggest that Sof exhibits potent antibacterial effects, not only against *S. aureus*, including drug-resistant strains such as MRSA, but also against Gram-negative bacteria like *E. coli*. Furthermore, Sof demonstrates an innovative mechanism of action that targets ribosomal proteins, thereby suppressing protein synthesis in *S. aureus*.

Sof’s antibacterial activity is notable for its potency against both drug-resistant strains and drug-sensitive of *S. aureus*. The IC_50_ values for Sof against *S. aureus* and MRSA were 10.42 and 11.13 μg/mL, respectively, indicating its more effectiveness in inhibiting bacterial growth than other natural products, such as madecassic acid, geraniol, baicalein and celastrol [48, 49]. This is particularly relevant in light of the growing need for new antibiotics capable of overcoming resistance to conventional treatments. Amoxicillin, a commonly used antibiotic, showed significantly higher IC_50_ values (93.36 μg/mL) against MRSA, highlighting the superior activity of Sof in this context. Moreover, the combination of Sof and amoxicillin yielded a synergistic effect, further emphasizing the potential of Sof in combination therapies for MRSA or multi-drug-resistant bacterial infections [50]. While traditional antibiotics typically target a specific bacterial process, Sof's ability to inhibit both Gram-negative and Gram-positive bacteria, such as *E. coli* and *S. aureus*, suggests a versatile mode of action. This broad-spectrum activity is consistent with the increasing demand for natural products that can target a wide range of pathogens, especially in the face of rising antibiotic resistance.

The mechanism of action of Sof is particularly noteworthy, as it diverges from conventional antibacterial agents. Our chemical proteomics analysis identified ribosomal proteins as the primary targets of Sof in *S. aureus*, with a particular focus on the rplB. It is worth noting that the ribosomal proteins, such as rplB, exhibits strong conservation in core structure and function across different bacterial strain (e.g. *H. pylori* and *S. aureus*), which is fundamental for ribosomal activity. Given that rplB serves as a key functional target of Sof, its structural and functional conservation across Gram-positive and Gram-negative bacteria likely contributes to the broad-spectrum antimicrobial activity of Sof. Additionally, Sof’s ability to covalently bind to ribosomal proteins via cysteine residues and inhibit protein synthesis represents an innovative approach to bacterial inhibition. The suppression of protein synthesis is a powerful mechanism, as the production of new proteins is essential for the growth and survival of bacterial cells [51]. Sof’s interaction with ribosomal proteins not only disrupts bacterial translation but also impairs other fundamental processes such as amino acid metabolism and glycolysis, as revealed by GO and KEGG pathway analyses. Other ribosome-targeting antibiotics, such as tetracyclines and macrolides, also inhibit protein synthesis, but their mechanisms of action are distinct [52]. For instance, macrolides bind to the 50S ribosomal subunit and prevent peptide elongation, while tetracyclines block the binding of aminoacyl-tRNA to the ribosome [53]. However, Sof stands out due to its unique chemical structure of electrophilic reactivity, which enables it to engage in Michael-type reactions with cysteine residues on bacterial proteins. Sof’s unique mode of action, involving covalent modification of ribosomal proteins, offers a novel strategy that could be exploited to develop new antibiotics that act differently from those currently in use.

In addition to ribosomal proteins, Sof was shown to suppress inflammatory responses in *S. aureus*-infected cells and animal models. Sof reduced the secretion of inflammatory cytokines, such as TNF-α, IL-1β, and IL-6, and alleviated lung injury in mice. Our and other’s prior studies has demonstrated that Sof inhibits inflammation response in LPS-induced Caco-2 cells and human gastric cancer cell [3, 16]. This anti-inflammatory effect adds another layer of therapeutic potential to Sof, as excessive inflammation is a hallmark of many bacterial infections, including pneumonia and sepsis [54]. By modulating both the bacterial load and the inflammatory response, Sof may offer a more holistic approach to treating infections, particularly those that lead to ALI and ARDS.

While this study highlights the promising antibacterial and anti-inflammatory efficacy of Sofalcone against *S. aureus* and MRSA, several limitations should be acknowledged. First, the pharmacokinetic properties of Sof, including absorption, distribution, metabolism, and excretion (ADME), remain uncharacterized. These parameters are critical for evaluating its bioavailability, dosing regimens, and potential toxicity in humans, which are prerequisites for clinical translation. Second, while Sof demonstrated efficacy in murine models, its therapeutic potential in larger animal models or human populations remains speculative. The biological differences between species and the complexity of human infections caused by multidrug-resistant pathogens necessitate further validation. Third, although ribosomal protein rplB was identified as a primary target, the possibility of additional molecular targets or off-site effects contributing to Sof’s antibacterial activity cannot be excluded. A more comprehensive exploration of Sof’s interactome may uncover supplementary mechanisms or unintended interactions. Fourth, the study did not systematically evaluate Sof’s ability to counteract bacterial resistance mechanisms, such as efflux pump activity or adaptive biofilm resilience, which are critical for long-term efficacy against evolving pathogens. Finally, while Sof synergized with amoxicillin in vitro, the broader applicability of combination therapies involving Sof and other antibiotics or natural agents remains untested. Addressing these limitations in future studies will be essential to fully realize Sof’s therapeutic potential and advance its development into a clinically viable antimicrobial agent.

In summary, Sof represents an innovative approach to combating bacterial infections, particularly those caused by *S. aureus*. Its unique mechanism of targeting ribosomal proteins and inhibiting protein synthesis sets it apart from conventional antibiotics. Furthermore, Sof’s broad-spectrum activity, coupled with its anti-inflammatory effects, makes it a promising candidate for the treatment of bacterial infections and associated inflammatory diseases. As antibiotic resistance continues to rise, the development of Sof and similar natural products could provide a valuable addition to the arsenal of antimicrobial agents.

## Materials and methods

### MIC determination experiment

Sof and the Sof probe (100 µg/mL) were serially diluted in Luria–Bertani (LB) liquid medium at ratios ranging from 1:2 to 1:256. Following dilution, 100 µL of each solution was added to individual wells in a 96-well cell culture plate. Each well was then inoculated with 10 µL of *S. aureus* at a concentration of 5 × 10^5^ CFU and incubated at 37℃ for 12 h. After incubation, bacterial growth was quantified by measuring the absorbance at 600 nm. The results were expressed as the minimum inhibitory concentrations (MICs).

### Checkerboard dilution assay

The synergistic antibacterial effect of Sof and amoxicillin was evaluated using a checkerboard assay adapted from established protocols [55]. Briefly, Sof and amoxicillin were combined at twice their individual MICs, followed by serial dilution to determine the MIC of the combined regimen. The calculation of ΣFIC is based on the formula: ΣFIC = FIC_sof_ + FIC_Amoxicillin_, where FIC_sof_ is defined as (MIC of Sof in combination / MIC of Sof alone), and FIC_Amoxicillin_ is defined as (MIC of Amoxicillin in combination / MIC of Amoxicillin alone). The interpretation of the results follows the standard criteria: synergy is defined as FIC ≤ 0.5, indifference as 0.5 < FIC < 2, and antagonism as FIC ≥ 2.

### In-gel fluorescence labeling experiment

In situ fluorescent labeling was performed as follows: *S. aureus* cultures were standardized in 150 mL LB broth (37℃, 18 h, OD_600_ = 1.0), pelleted via centrifugation (8,000 × g, 10 min), and resuspended in 1 mL fresh LB medium. Bacterial suspensions were supplemented with Sof-P or vehicle (DMSO) for 8-h exposure at 37℃, followed by triple PBS washing. Cell lysis was achieved using 100 μL BeyoLytic™ reagent, with clarified lysates obtained through centrifugation (12,000 × g, 15 min). Protein concentrations were quantified via BCA assay (Pierce™), with 200 μg aliquots subjected to copper(I)-catalyzed azide-alkyne cycloaddition (CuAAC) under optimized conditions: 3 μL TCEP (50 mM aqueous), 3 μL CuSO_4_ (50 mM aqueous), 9 μL TBTA (10 mM DMSO), and 0.9 μL TAMRA-azide (10 mM DMSO) in 100 μL reaction volume. The mixture underwent rotational agitation (800 rpm, 25℃, 2 h), followed by protein precipitation using ice-cold acetone (-80℃, 30 min) and centrifugation (15,000 × g, 10 min). The protein pellets were dissolved in 50 µL of 1 × loading buffer, denatured at 95℃ for 5 min, and resolved on 12% SDS-PAGE gels. The labeled proteins were visualized using a laser scanner (Azure Sapphire RGBNIR, USA) and stained with Coomassie Brilliant Blue (CBB).

In the *in-situ* fluorescent labeling competition experiments, *S. aureus* was pretreated with Sof for 4 h, followed by incubation with Sof-P for 8 h. After treatment, protein extraction, click chemistry, and electrophoretic separation were performed as described above. The labeled proteins were analyzed using a laser scanner (Azure Sapphire RGBNIR, USA) and stained with CBB.

### Activity-based protein profiling experiment

Competitive chemoproteomic profiling was conducted by treating *S. aureus* suspensions with Sof-P (25 µM) with or without tenfold molar excess of unmodified Sof for 8 h. Post-treatment, bacterial lysates were subjected to copper(I)-catalyzed biotin-azide conjugation, followed by acetone precipitation (-80℃, 30 min) and resolubilization in 1.2% SDS. Streptavidin magnetic beads (50 µL slurry) were incubated with samples (4 h, RT) for target enrichment, with sequential washes using: (i) 1% SDS, (ii) 0.1% SDS, and (iii) 6 M urea/PBS. Captured proteins underwent disulfide reduction (10 mM DTT, 56℃, 30 min) and cysteine alkylation (55 mM IAA, RT, 30 min), followed by tryptic digestion (37℃, 16 h). Peptides were acidified, desalted via C18 StageTips, and labeled with TMT10-plex reagents per manufacturer’s protocol. Multiplexed samples were analyzed by nanoflow LC–MS/MS (Orbitrap Fusion Lumos, Thermo Scientific) using a 120-min gradient (2–35% acetonitrile/0.1% formic acid).

### Protein labeling assay of rplB by Sof-P

To study the interaction between Sof and rplB, five experimental groups were set up: a blank control group, the Sof-P (1, 2, 5, 10 μM) group, with each group containing 5 μg of pure rplB protein. The corresponding concentrations of Sof-P were added to the experimental groups, followed by incubation at 37℃ with shaking at 800 rpm for 1 h. The click reaction was performed for 1 h. After the reaction, the proteins were denatured, and SDS-PAGE was performed. Fluorescence observation was followed by staining with CBB.

### Competition labeling assay of rplB by Sof-P

Five groups were established for the competition experiment: a blank control group, a Sof-P group, a Sof competition group (5 × and 10 × concentrations), and an IAA competition group, with each group containing 5 μg of pure rplB protein. The corresponding concentrations of drugs were added to the Sof competition and IAA competition groups, and the samples were incubated at 37℃ with shaking at 800 rpm for 2 h. Subsequently, 10 μM of Sof-P was added to all groups except the control group, and incubation was continued for 1 h. The click reaction was carried out for 1 h, and upon completion, the proteins were denatured and analyzed by SDS-PAGE. Fluorescence was observed and the samples were stained with CBB.

### Cellular thermal shift assay

CETSA was employed to investigate the interaction between sofalcone and rplB. Briefly, the pure rplB protein sample was divided into two equal portions. One portion was incubated with sofalcone, and the other with DMSO, at 37℃ for 1 h. The mixtures were then transferred to PCR tubes and subjected to heating at specific temperatures (42, 47, 52, 57, 62, 67, and 72℃) for 3 min using a thermal cycler (Applied Biosystems, USA). After centrifugation, the supernatants were analyzed by SDS-PAGE, and the proteins were stained with CBB to assess binding.

### Microscale thermophoresis assay

Microscale thermophoresis (MST) was employed to quantitatively assess the interaction between rplB and Sof. The binding affinity was measured using a Monolith NT.115 (Nano Temper Technologies, Munich, Germany). Briefly, recombinant *S. aureus* rplB protein was labeled with fluorescence using the Monolith His-Tag labeling kit (Nano Temper Technologies, Munich, Germany). Sof was then serially diluted and mixed with the rplB solution (50% v/v). The mixtures were loaded into capillaries and analyzed using MST at 40% power. The data were processed using MO Affinity Analysis software v2.3.

### In-gel fluorescence labeling of newly synthesized protein experiment

Activated *S. aureus* was cultured in 150 mL LB liquid medium at 37℃ for 18 h until reaching an OD_600_ of 1.0. The bacteria were then harvested by centrifugation at 8000 rpm and washed three times before being resuspended in 20 mL of M9 minimal medium (excluding methionine). The bacteria were incubated at 37℃ for 30 min to deplete intracellular methionine reserves. Subsequently, 50 µM of AHA (Click Chemistry Tools) was added to each experimental group, both in the absence and presence of Sof. After incubation for varying durations (15 min, 30 min, 1 h and 2 h), the bacteria were collected and lysed with 100 µL of bacterial lysis solution. Protein concentrations were determined and adjusted to ensure consistency across all experimental groups. The click chemistry reaction was performed similarly to previous protocols, with the modification that TAMRA-azide was replaced by TAMRA-alkynyl for fluorescent labeling.

For concentration-dependent labeling experiments, *S. aureus* was initially treated with DMSO, Sof (5, 10, 20, 40 µg/mL), and CHX (50 µM) in the absence of 50 µM AHA for 1 h. Subsequently, protein extraction, click chemistry reactions, and electrophoretic separation were conducted following the methods described previously, and visualized accordingly.

### Newly synthesized protein identification assay

The bacterial or BEAS-2B cell suspension was treated with Sof (20 µg/mL) in the presence of 50 µM AHA for 1 h. Proteins were extracted as described previously and conjugated with biotin-alkynyl through a click chemistry reaction. The samples were then precipitated with pre-chilled acetone and re-dissolved in 1.2% SDS. Streptavidin beads (50 µL) were added and incubated for 4 h at room temperature. After incubation, the beads were washed sequentially with 1% SDS, 0.1% SDS, and 6 M urea in PBS. The samples underwent reduction and alkylation using DTT and IAA. The proteins bound to the beads were digested into peptides using trypsin. The resulting peptides were collected by centrifugation, desalted with C18 columns, and subjected to Label-Free Quantification (LFQ) proteomics analysis using LC–MS/MS (Orbitrap Fusion Lumos, Thermo, USA).

### Bioinformatic analysis

The abundance changes based on criteria of absolute fold change ≥ 1.5 and a P-value (FDR) < 0.05. Subsequently, a volcano plot was generated using the bioladder website (https://www.bioladder.cn). The proteome function database of *S. aureus* (Taxonomy ID 1279) was downloaded from the website of Uniprot (https://www.uniprot. org/). The biological process and KEGG pathway enrichment were conducted for functional profile visualization using Omicshare tool (https://www.omicshare.com/).

### Mice experiment

In this study, animal experimental protocols were approved by the Ethics Committee of China Academy of Chinese Medical Sciences (2023B299). Male BALB/c mice (aged 7–8 weeks) were procured from Vital River Laboratory Animal Technology and housed under standard environmental conditions. A total of 30 mice were randomly divided into 5 groups, with 6 mice per group. During a 3-day acclimatization period, the mice were provided with diets and water acclimated to the humidity, temperature, ventilation, and light in the sterile animal facility. Due to the limitations of bacterial infection animal models, bacteria typically survive in mice for only 48 h post-infection, with peak colonization occurring at 24 h [56–58]. Therefore, a combined preventive and therapeutic administration approach was employed to assess the in vivo antibacterial effects of Sof, which is also a commonly used and accepted method in pharmacological evaluations of antibacterial drugs [58]. The mice received daily administration of the following treatments for 4 days: 0.5% sodium carboxymethylcellulose (CMC-Na, control, model), 0.5% CMC-Na + 25 mg/kg Sof, 0.5% CMC-Na + 50 mg/kg Sof, and LPS + 100 mg/kg Sof. An acute lung injury model infected by bacteria was induced on the 5th day through intranasal administration of *S. aureus* (OD_600_ = 1.0). Finally, at 24 h after intranasal administration of *S. aureus*, blood, lung tissues, and bronchoalveolar lavage fluid were collected for subsequent assays. Tissue allocation protocols and sample exclusion criteria were implemented to ensure analytical validity. Final sample sizes per assay are annotated in the respective figure legends.

### Statistical analysis

Data analysis was performed using GraphPad Prism 8.0 software (San Diego, CA, USA). All data are expressed as mean ± standard error and were analyzed using an unpaired one-tailed Student’s t-test. *p*-value of less than 0.05 was considered statistically significant.

## Supplementary Information

Supplementary Material 1. FigureS1-S9, and additional materials and methods are included in the Supplementary Material 1.

Supplementary Material 2. Protein target list of Sof is included in the Table S1. The list of newly synthesized proteins inhibited by Sof is included in the Table S2. 

## Data Availability

The datasets and materials supporting our findings in the current study are available from the corresponding authors on reasonable request.
