# Supplementary material for: Chemoproteomics unveils Sofalcone targeting ribosomal proteins to inhibit protein synthesis in Staphylococcus aureus
Source: Mol Biomed. 2025 May 23;6:32. doi: 10.1186/s43556-025-00269-4 (PMC12102032; doi:10.1186/s43556-025-00269-4)
Supplement: Supplementary file 1 — Supplementary Material 1. FigureS1-S9, and additional materials and methods are included in the Supplementary Material 1. [file 43556_2025_269_MOESM1_ESM.docx]

**Chemoproteomics unveils Sofalcone targeting ribosomal proteins to inhibit protein synthesis in *Staphylococcus aureus***

Lirun Zhou^a,#^, Ying Zhang^b,#^, Ruishen Zhuge^d,#^, Liqiong Wu^e,#^, Zheng Chu^a^, Ang Ma^a^, Peng Gao^a^, Yin Kwan Wong^f^, Junzhe Zhang^a^, Xin Peng^g,*^, Peili Wang^h,*^, Jigang Wang^a,b,c,*^, and Huan Tang^a,*^

^a^ State Key Laboratory for Quality Ensurance and Sustainable Use of Dao-di Herbs, Artemisinin Research Center, and Institute of Chinese Materia Medica, China Academy of Chinese Medical Sciences, Beijing 100700, China.

^b^ Department of Pulmonary and Critical Care Medicine, Shenzhen Institute of Respiratory Diseases, Guangdong Provincial Clinical Research Center for Geriatrics, Shenzhen Clinical Research Center for Geriatrics, Shenzhen People's Hospital, The First Affiliated Hospital, Southern University of Science and Technology, Shenzhen, Guangdong 518020, China.

^c^ State Key Laboratory of Antiviral Drugs, School of Pharmacy, Henan University, Kaifeng 475004, China.

^d^ Peking University School and Hospital of Stomatology, National Center for Stomatology, National Clinical Research Center for Oral Diseases, National Engineering Research Center of Oral Biomaterials and Digital Medical Devices, Beijing 100081, China.

^e^ Department of Pathology, Guangzhou First People’s Hospital, Guangzhou 510180, China.

^f^ Department of Physiology, National University of Singapore, Singapore, 117543, Singapore.

^g^ Ningbo Municipal Hospital of TCM Affiliated Hospital of Zhejiang Chinese Medical University, Ningbo 315010, China.

^h^ National Clinical Research Center for Chinese Medicine Cardiology, Xiyuan Hospital, China Academy of Chinese Medical Sciences, Beijing 100091, China.

^#^ These authors contributed equally to this work.

^*^ Corresponding authors. E-mail addresses: htang@icmm.ac.cn (H. Tang); jgwang@icmm.ac.cn (J. Wang); qiexuxing0721@163.com (P. Wang); pengx@nit.zju.edu.cn (X. Peng).

**
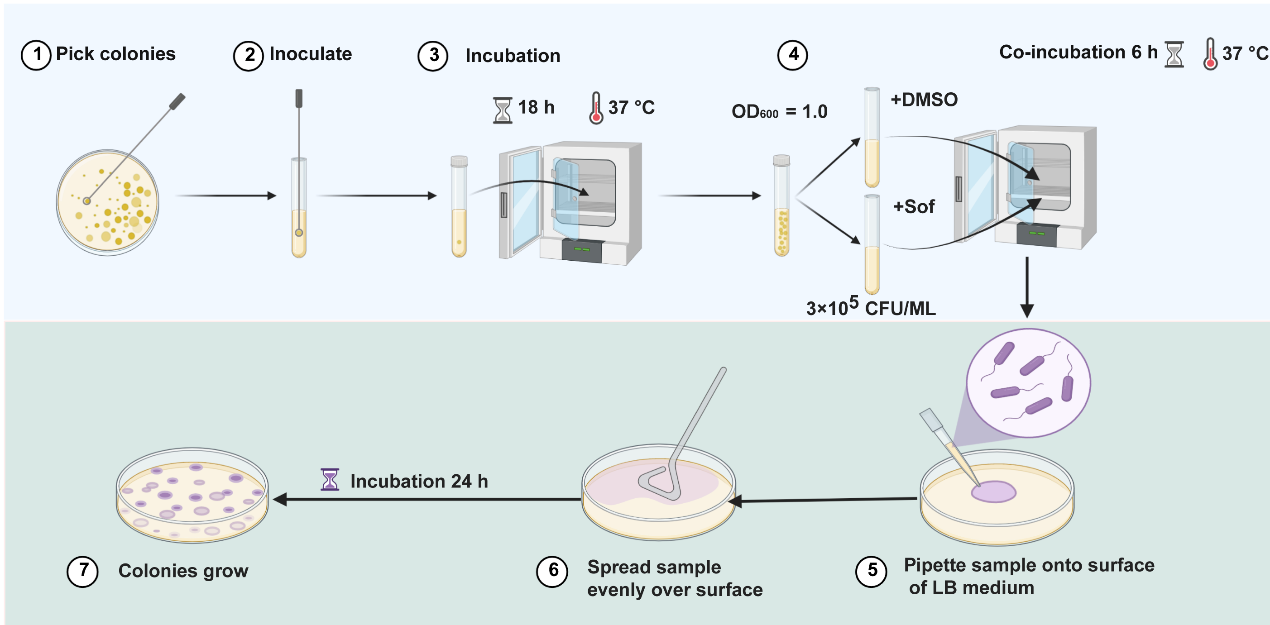
**

**Figure S1.** Flowchart of *S. aureus* colony measured by the plate colony counting method in the absence and presence of Sof.


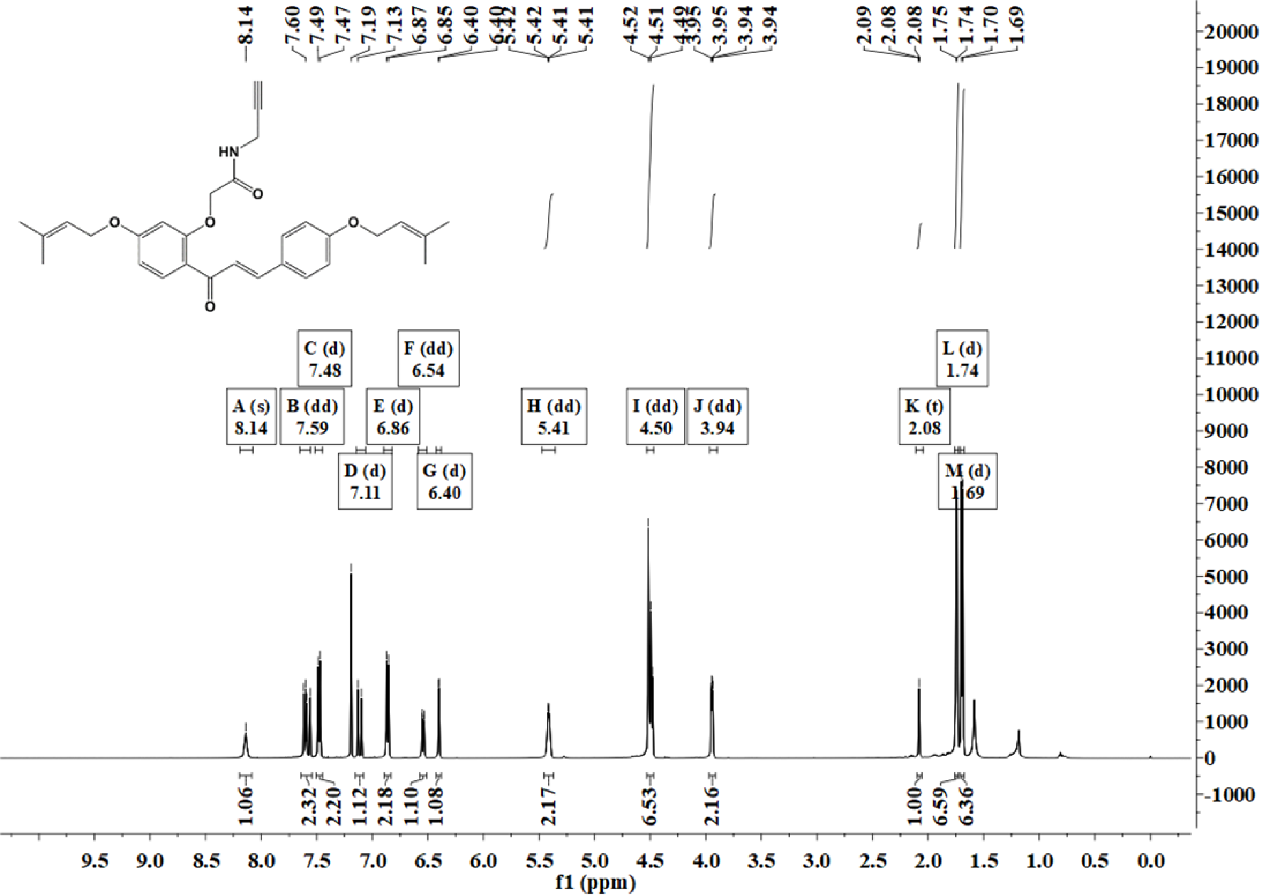


**Figure S2.** ^1^H-nuclear magnetic resonance spectrum of Sof-P.


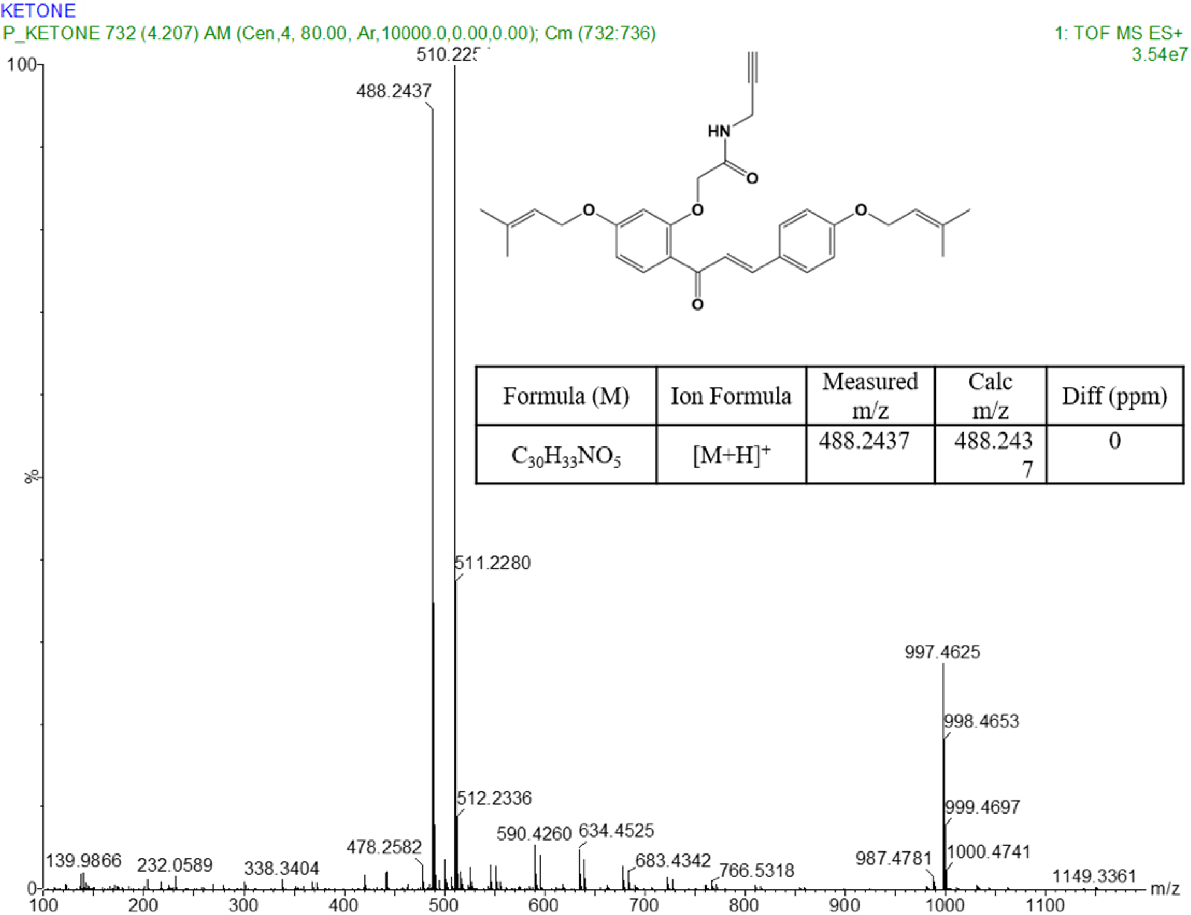


**Figure S3.** High-resolution mass spectrum of Sof-P.

**
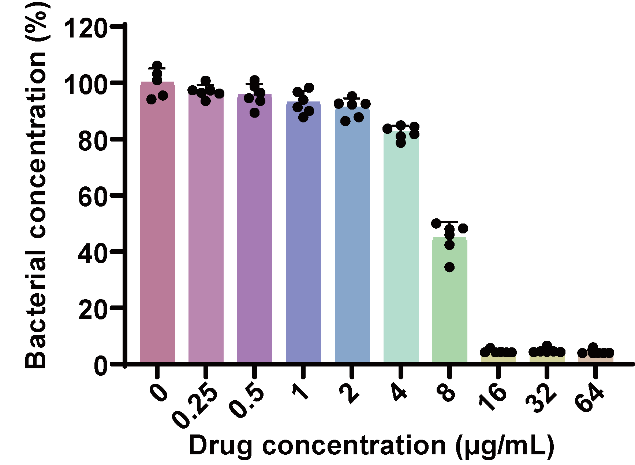
**

**Figure S4.** Bacterial concentration of *Staphylococcus aureus* (*S. aureus*) following 12 h of treatment with Sof-P at different concentrations.

**
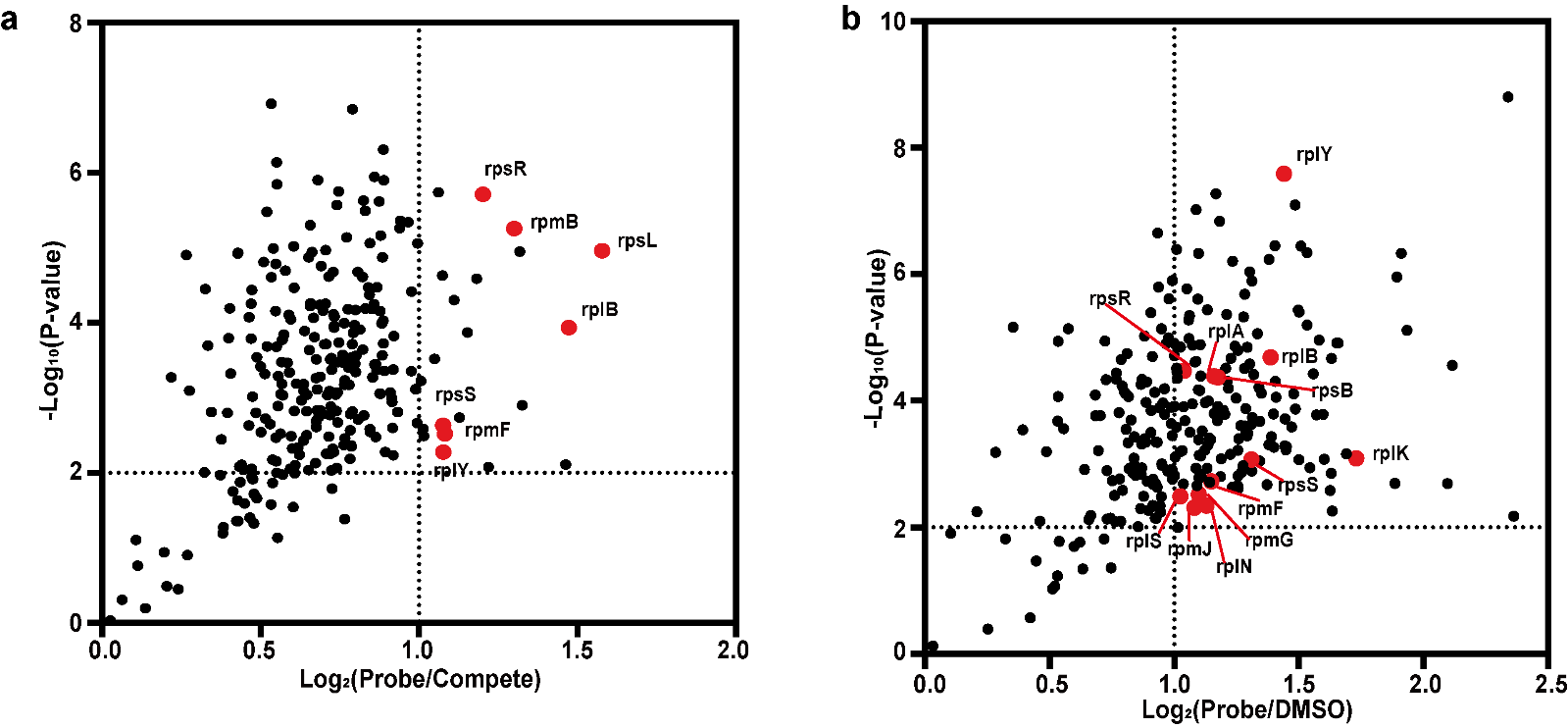
**

**Figure S5.** (a) Volcano plot of protein targets in the Sof-P group versus the competition group. (b) Volcano plot of protein targets in the Sof-P group versus the control group.

**
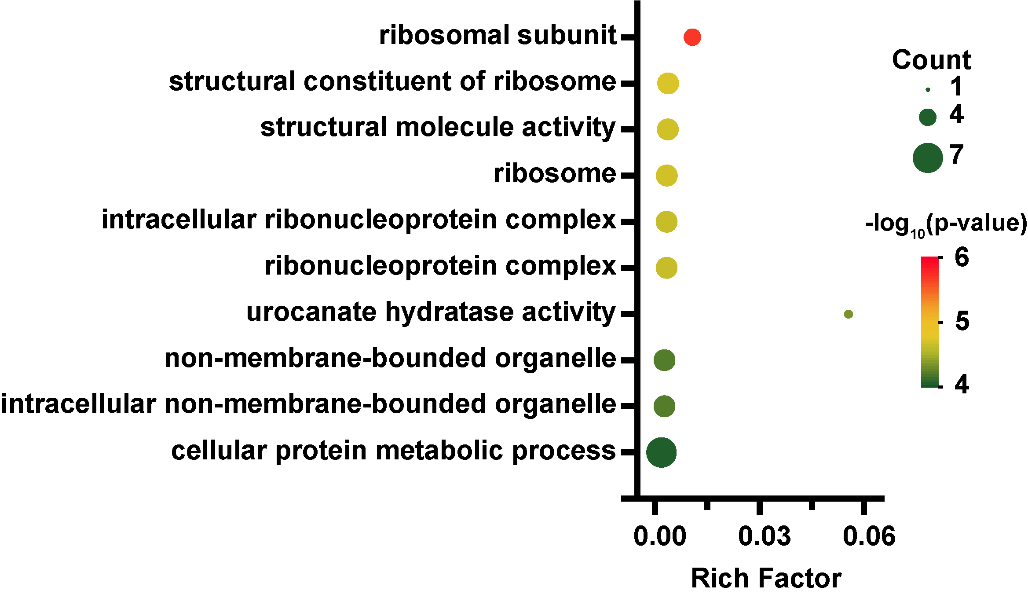
**

**Figure S6.** Gene ontology enrichment analysis of Sof-targeted proteins.

**
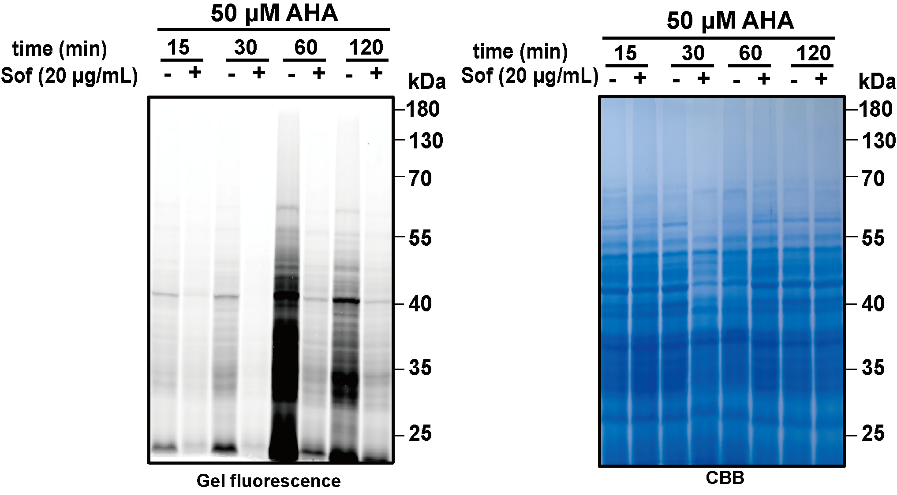
**

**Figure S7.** In-gel fluorescent imaging of proteins in *S. aureus* by AHA metabolic labeling after Sof treatment for different times. CBB, Coomassie brilliant blue.

**
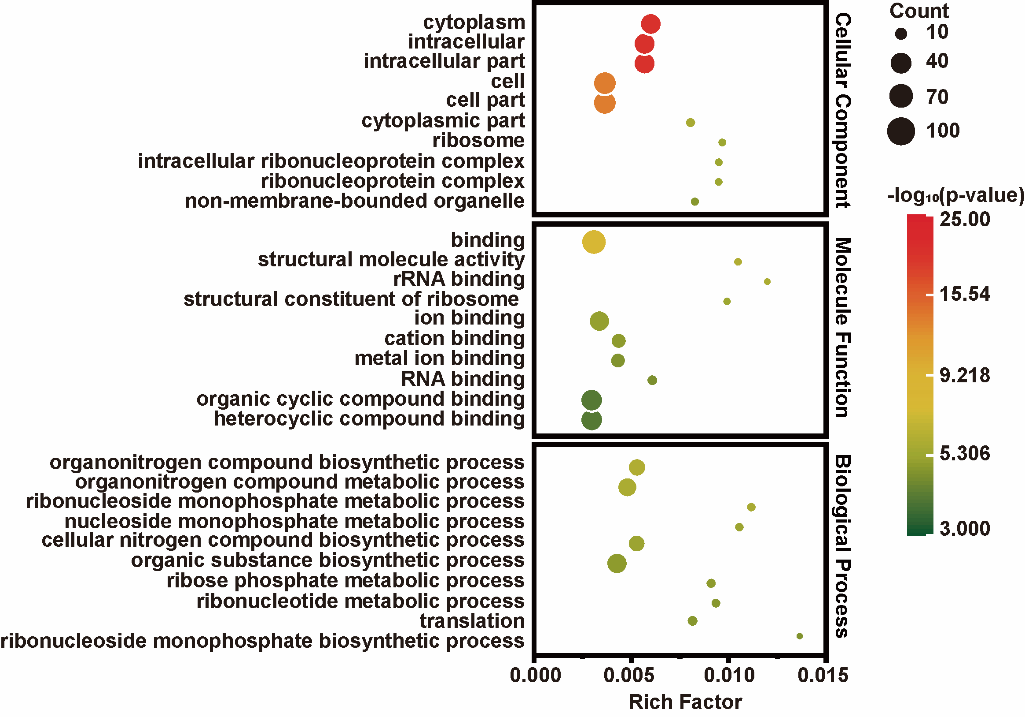
**

**Figure S8.** Gene ontology enrichment analysis of proteins whose *de novo* synthesis inhibited by Sof in terms of cellular component, molecule function, and biological processes.

**
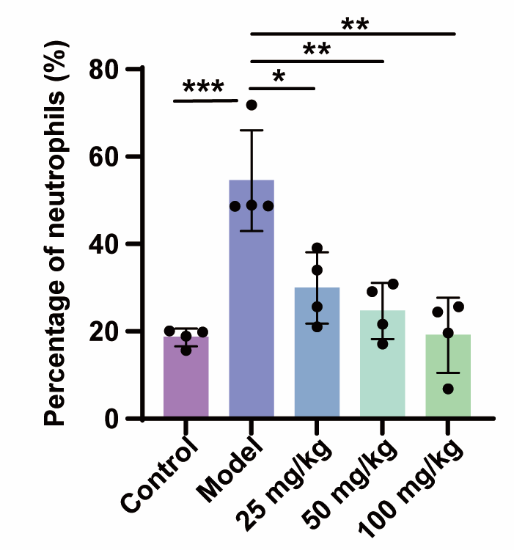
**

**Figure S9.** Percentage of neutrophils in peripheral anticoagulation blood of *S. aureus*-infected mice before and after Sof treatment. Data are expressed as the mean ± SEM; *p < 0.05, **p < 0.01, ***p < 0.001.

**Materials and methods**

**Reagents**

Sofalcone (Sof, purity ≥ 97%) was procured from Bide Pharmatech Co., Ltd. (Shanghai, China). L-azidohomoalanine (AHA) was supplied by Click Chemistry Tools (USA). Tris(2-carboxyethyl)phosphine hydrochloride (TECP), TAMRA-azide, Biotin-azide, Tris[(1-benzyl-1H-1,2,3-triazol-4-yl)methyl]amine (TBTA), Biotin-alkynyl, Copper (II) sulfate (CuSO_4_), and TAMRA-alkynyl were obtained from Sigma-Aldrich (USA). High-capacity the Pierce™ Quantitative Fluorometric Peptide Assay Kit, NeutrAvidin agarose beads, the TMT10plex reagent kit, sequencing-grade modified trypsin, and tetraethylammonium bromide (TEAB) were purchased from Thermo Fisher Scientific (USA). Primary antibodies targeting IL-6, TNF-α, and IL-1β were sourced from Proteintech (China), while the anti-β-actin antibody was acquired from Affinity Biosciences (China).

**Evaluation of antibacterial effect of natural products**

Twenty-four drugs, including Sof, were diluted to a concentration of 30 μg/mL using LB medium. Subsequently, 100 µL of each drug dilution was added to individual wells in a 96-well cell culture plate. The wells were then inoculated with 10 µL of *S. aureus* at a concentration of 5 × 10^5^ colony-forming units (CFU) and incubated at 37 ℃ for 12 hours. After incubation, bacterial concentration was quantified by measuring the absorbance at 600 nm.

**Synthesis of Sof probe (Sof-P)**

The Sof probe was synthesized following our previously published procedure [1]. L-amino-2-propargyl (0.4 mmol, 22.03 mg) and Sof (0.2 mmol, 97.45 mg) were dissolved in 2 mL of anhydrous CH_2_Cl_2_. The reaction was initiated by adding the catalysts 1-hydroxybenzotriazole (HOBt, 0.6 mmol, 81.08 mg), N, N-diisopropylethylamine (DIPEA, 0.6 mmol, 77.55 mg), and 1-(3-dimethylaminopropyl)-3-ethylcarbodiimide hydrochloride (EDCI, 0.6 mmol, 115.02 mg). The mixture was stirred at ambient temperature for 24 hours. After the reaction reached completion, the solvent was removed under reduced pressure, and the crude product was purified using silica gel column chromatography to afford Sof-P as a white solid in 83% yield. The ^1^H NMR spectrum (500 MHz, CDCl_3_) showed 7.59 (dd, *J* = 16.9, 12.2 Hz, 2H) , 3.94 (dd, *J* = 5.4, 2.4 Hz, 2H), 6.54 (dd, *J* = 8.6, 1.9 Hz, 1H), 2.08 (t, *J* = 2.4 Hz, 1H) , 7.48 (d, *J* = 8.7 Hz, 2H), 6.86 (d, *J* = 8.6 Hz, 2H) , 1.69 (d, *J* = 6.4 Hz, 6H), δ 8.14 (s, 1H), 6.40 (d, *J* = 1.9 Hz, 1H), 5.41 (dd, *J* = 3.2, 1.4 Hz, 2H), 4.50 (dd, *J* = 13.3, 5.4 Hz, 6H), 1.74 (d, *J* = 4.8 Hz, 6H), 7.11 (d, *J* = 15.7 Hz, 1H). High-resolution mass spectrometry (HRMS) analysis gave m/z: [M+H]^+^ calculated for C_30_H_33_NO_5_ as 488.2437, with an observed value of 488.2437.

**Plate colony counting method**

Sof and Sof-P were diluted in LB liquid medium to predetermined concentrations, and each medium was inoculated with 10 µL of *S. aureus* at a concentration of 5 × 10^5^ Colony-Forming Units‌ (CFU). After an initial incubation at 37 ℃ for 6 hours, the samples were further diluted 1:1000, and 100 µL of the diluted solution was plated onto LB solid media. The plates were incubated at 37 ℃ for an additional 24 hours. Finally, the number of colonies was counted.

**Cell and S. *aureus* culture**

HCoEpic and BEAS-2B cell lines were obtained from the Chinese Academy of Medical Sciences (Beijing, China). BEAS-2B cell lines used in this study were authenticated through short tandem repeat (STR) analysis by the Xiamen Yimo Biotechnology Co., Ltd. And HCoEpic cell lines used in this study were purchased from Tongpai (Shanghai) Biotechnology Co., Ltd as quality-controlled primary cells with accompanying quality inspection reports. Additionally, mycoplasma inhibitors were employed during the culture process, and regular checks were conducted to ensure the absence of mycoplasma contamination. Both cell types were maintained in high-glucose DMEM (Corning, USA) supplemented with 10% FBS (Corning, USA) and 100 IU/mL penicillin-streptomycin (Thermo Fisher, USA) at 37 ℃ under a humidified atmosphere containing 5% CO_2_.

*S. aureus* (ATCC 6538) was obtained from Shanghai Luwei Technology Co., Ltd. and preserved at -80 ℃. For activation, a 100-µL aliquot of the bacterial suspension was evenly spread on LB agar plates (containing 1 g NaCl, 0.5 g yeast extract, 1 g tryptone, and 1.5 g agar per 100 mL) and incubated at 37 ℃ for 24 hours. Smooth colonies were then inoculated into 30 mL of LB broth and cultured in a shaker incubator at 37 ℃ and 120 rpm for 18 hours. Bacteria at the stationary growth phase (OD_600_ = 1.0) were harvested for subsequent experiments.

**Cell viability**

HCoEpic and BEAS-2B cells were plated into 96-well plates at a density of 1.0 × 10⁴ cells per well and cultured for 24 hours. Subsequently, the cells were exposed to different concentrations of Sof for an additional 12 hours. Cell viability was determined using the Cell Counting Kit-8 (CCK-8) (Dojindo, Japan) following the manufacturer’s protocol. Absorbance at 450 nm was recorded with a multimode plate reader (PerkinElmer, USA).

**Morphology observation of bacterial by SEM and TEM**

For TEM analysis, the samples were washed three times with PBS, and a 10 µL aliquot was placed onto a glow-discharged copper grid. The samples were then stained with 0.4% uranyl acetate for 20 seconds before imaging [2].

For SEM analysis, the samples were washed with PBS and fixed overnight with 2.5% glutaraldehyde. After washing three times with PBS, the samples were sequentially dehydrated with 50%, 70%, 85%, and 95% ethanol, with one wash for each concentration. The samples were then dehydrated with absolute ethanol three times for 15 minutes each. After supercritical drying with carbon dioxide, the samples underwent gold sputtering treatment and were observed under a scanning electron microscope [2].

**The invasion and adhesion of** ***S. aureus* to BEAS-2B cells**

*S. aureus* and Sof incubation for 4 hours. Afterward, bacteria were harvested, resuspended in DMEM, and used to infect BEAS-2B cells at a multiplicity of infection (MOI) of 100 (bacteria-to-cell ratio of 100:1) for 3 hours.

For the adhesion assay, the cell monolayers were rinsed three times with PBS. Then, 1 mL of 0.2% Triton X-100 was added, and the contents were mixed by pipetting up and down ten times. The lysates were incubated at 37 ℃ for 10 minutes, diluted 1000-fold, and plated on agar for colony counting [3].

For the invasion assay, the culture medium was replaced with 1 mL of antibiotic-containing medium and incubated for 1 hour. The invasion detection method is the same as adhesion assay [3].

**Effect of Sof on the biofilm formation of *S. aureus*.**

Overnight-cultured *S. aureus* was diluted to a final concentration of 1.0 × 10⁷ CFU/mL. Experimental groups included a bacterial control, a blank control, and seven treatment groups. Each group received 200 µL of the bacterial suspension. Sof was added to the treatment groups at difference concentrations, while the control groups were treated with an equivalent volume of DMSO. All samples were incubated at 37 ℃ for 48 hours. Following incubation, the wells were carefully aspirated, washed twice with sterile distilled water, and air-dried inside a biosafety cabinet. Each well was stained with 200 µL of 1% crystal violet for 15–20 minutes. Excess stain was removed, and the wells were rinsed with 300 µL of sterile distilled water. The plates were inverted on filter paper to drain residual water and dried at either room temperature or 37 ℃. To solubilize the crystal violet, 200 µL of 33% acetic acid was added to each well and incubated at 37 ℃ for 30 minutes. The absorbance was read at 590 nm [4].

**Effect of Sof on the inflammation response of BEAS-2B cells induced by *S. aureus***

The drug treatment is the same as invasion and adhesion assay. The infection was carried out for 24 hours. Nitric oxide (NO) levels in the supernatants were determined using the Griess reagent following the manufacturer’s instructions (Solarbio, China). The concentrations of TNF-α (ml064303), IL-1β (ml058059), and IL-6 (ml058097) in the cell culture supernatants were measured according to the protocols provided by Shanghai Enzyme-linked Biotechnology Co., Ltd.

**Dead/Live fluorescence staining assay**

The OD_600_ of *S. aureus* was adjusted to 0.6. A bacterial control group and three experimental groups, each containing 5 mL of bacterial suspension, were established. The corresponding concentrations of Sof were added to each treatment group, while the control group received the appropriate volume of DMSO. The groups were incubated at 37 ℃ with continuous shaking at 120 rpm for 12 hours. After incubation, lLive/dead cell staining was performed using the Calcein-AM/PI kit (Solarbio, CAT: CA1630) according to the manufacturer’s instructions. Fluorescent images were captured using a confocal microscope.

**Growth Curve and Killing Curve**

Bacterial suspensions of *S. aureus* were prepared in LB broth with OD_600_ values adjusted to 0.2 and 0.6 for growth curve and killing curve experiments, respectively. The suspensions were incubated with various concentrations of Sof at 37 ℃ under continuous shaking at 160 rpm. At designated time intervals, the optical density at 600 nm (OD_600_) of 1-mL samples was measured using a micro-spectrophotometer [5].

**Cell toxicity of *S. aureus* on BEAS-2B cells**

The concentration of *S. aureus* was adjusted to OD_600_ = 0.6. Various concentrations of Sof (0, 0.25, 0.5, 1 μg/mL) were added to the bacterial suspension, which was incubated for 4 hours. The bacteria were then collected, resuspended in DMEM, and used to infect BEAS-2B cells at a MOI of 100. The infection was allowed to proceed for 12 hours. Cytotoxicity was assessed using the CCK-8 assay.

**Histopathologic analysis**

Mouse lung and liver tissues were fixed in 4% paraformaldehyde, followed by paraffin embedding and sectioning. The tissue sections were stained with hematoxylin and eosin (H&E) to evaluate pathological alterations. After dehydration and mounting, images of the sections were captured using a digital slide scanner (Nanozoomer-SQ, Hamamatsu Photonics) [6].

For immunohistochemistry, the tissue sections were incubated with primary antibodies against IL-6, TNF-α, and IL-1β overnight at 4℃. Following this, the sections were incubated with the appropriate secondary antibody at room temperature for 2 hours. After dehydration and mounting, the sections were imaged using the digital slide scanner.

For western blotting, total proteins were extracted from the lung tissues using RIPA lysis buffer (Beyotime, China) supplemented with a 1× protease inhibitor cocktail (ThermoFisher, USA). The proteins were separated on 12% SDS-PAGE gels, then transferred to polyvinylidene fluoride (PVDF) membranes for 1.5 hours at 120 V. The membranes were blocked with 5% bovine serum albumin (BSA) and incubated overnight at 4℃ with the appropriate primary antibodies. The sections were then incubated with the corresponding secondary antibody at room temperature for 2 hours. Protein bands were visualized by chemiluminescence (ECL, Thermo Fisher, USA) and semi-quantified using ImageJ software.

**Determination of colony forming units**

Bacterial counts in infected bronchoalveolar lavage fluid (BALF) were performed. Animals were euthanized by cervical dislocation on day 6. The BALF were diluted and cultured on LB medium at 37℃ for 24 hours, and the number of colonies in each group was recorded [7].

**Cytokine and blood routine assays**

On day 6, blood was collected into centrifuge tubes coated with sodium heparin from the sacrificed animal groups. Whole blood was diluted after mixing for routine blood tests, and then plasma was separated. The concentrations of TNF-alpha (ml002095), IL-1β (ml098416), IL-6 (ml063159), and CRP (ml038364) in plasma were measured according to the manufacturer’s instructions (Shanghai Enzyme-linked Biotechnology Co., Ltd.).

**References**

1. Yang T, Liu D, Li Y, Zhang Y, Zhu Y, Zhang J, et al. Chemoproteomics reveals Sofalcone inhibits the inflammatory response of Caco-2 cells by covalently targeting HMGB1. Chem Commun (Camb). 2023;59(58):8981-4. <https://doi.org/10.1039/d3cc00577a>

2. Zhang S, Qu X, Tang H, Wang Y, Yang H, Yuan W, et al. Diclofenac Resensitizes Methicillin-Resistant S. aureus to β-Lactams and Prevents Implant Infections. Adv Sci (Weinh). 2021;8(13):2100681. https://doi.org/10.1002/advs.202100681

3. Prystopiuk V, Feuillie C, Herman-Bausier P, Viela F, Alsteens D, Pietrocola G, et al. Mechanical Forces Guiding S. aureus Cellular Invasion. ACS Nano. 2018;12(4):3609-22. https://doi.org/10.1021/acsnano.8b00716

4. Yuan Z, Lin C, He Y, Tao B, Chen M, Zhang J, et al. Near-Infrared Light-Triggered Nitric-Oxide-Enhanced Photodynamic Therapy and Low-Temperature Photothermal Therapy for Biofilm Elimination. ACS Nano. 2020;14(3):3546-62. https://doi.org/10.1021/acsnano.9b09871

5. Yuan Z, Wang J, Qu Q, Zhu Z, Xu M, Zhao M, et al. Celastrol Combats Methicillin-Resistant S. aureus by Targeting Δ(1) -Pyrroline-5-Carboxylate Dehydrogenase. Adv Sci (Weinh). 2023;10(25):e2302459. https://doi.org/10.1002/advs.202302459

6. Xu W, Tian K, Li X, Zhang S. IL-9 blockade attenuates inflammation in a murine model of methicillin-resistant S. aureus pneumonia. Acta Biochim Biophys Sin (Shanghai). 2020;52(2):133-40. <https://doi.org/10.1093/abbs/gmz149>

7. Shatzkes K, Singleton E, Tang C, Zuena M, Shukla S, Gupta S, et al. Predatory Bacteria Attenuate Klebsiella pneumoniae Burden in Rat Lungs. mBio. 2016;7(6). https://doi.org/10.1128/mBio.01847-16
